# Facet Dependence of the Oxygen Evolution Reaction on Co_3_O_4_, CoFe_2_O_4_, and Fe_3_O_4_ Epitaxial Film Electrocatalysts

**DOI:** 10.1021/jacs.3c13595

**Published:** 2024-05-08

**Authors:** Earl Matthew Davis, Arno Bergmann, Helmut Kuhlenbeck, Beatriz Roldan Cuenya

**Affiliations:** Department of Interface Science, Fritz Haber Institute of the Max Planck Society, Faradayweg 4–6, 14195 Berlin, Germany

## Abstract

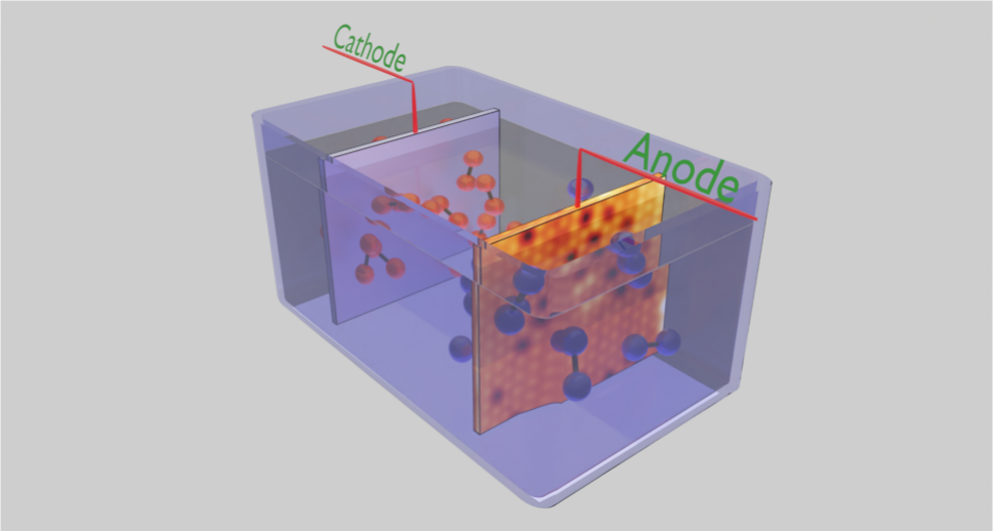

The main obstacle for the electrocatalytic production of “green hydrogen” is finding suitable electrocatalysts which operate highly efficiently over extended periods of time. The topic of this study is the oxygen evolution reaction (OER), one of the half-reactions of water splitting. It is complex and has intricate kinetics, which impairs the reaction efficiency. Transition metal oxides have shown potential as electrocatalysts for this reaction, but much remains unknown about the atomic scale processes. We have investigated structure–composition–reactivity correlations for Co_3_O_4_, CoFe_2_O_4_, and Fe_3_O_4_ epitaxial thin-film electrocatalysts exposing either the (001) or (111) surface facets. We found that for Co_3_O_4_, the (001) facet is more reactive, while for the other oxides, the (111) facet is more active. A Tafel-like evaluation reveals systematically smaller “Tafel” slopes for the (001) facets. Furthermore, the slopes are smaller for the iron-containing films. Additionally, we found that the oxyhydroxide skin layer which forms under OER reaction conditions is thicker on the cobalt oxides than on the other oxides, which we attribute to either a different density of surface defects or to iron hindering the growth of the skin layers. All studied skin layers were thinner than 1 nm.

## Introduction

As the world is trying to switch to sustainable energy sources, fluctuating supplies require an efficient method of energy storage and transport, making energy available when it is required and where it is required. One potential route is the electrocatalytic splitting of water. The limiting factor of this reaction is the low efficiency of the oxygen evolution reaction (OER).^1,2^

To overcome this issue and make this method of energy storage and transport viable for large-scale use, efficient and low-cost catalysts are being sought. Oxides of several abundant transition metals have shown promise in this regard, in particular oxide spinels containing Co, Ni, and Fe.^2−4^ Despite their promising electrocatalytic performance, much is unknown about the surfaces of these materials under reaction conditions and about their interaction with the electrolyte when a potential is applied.

Catalytic studies of realistic systems are often hampered by the catalyst’s complexity. A common way to handle this issue is to study simplified systems, so-called “model catalysts”. Epitaxial thin films can serve as such: they have flat surfaces, a homogeneous composition, and a well-defined structure, which makes them much simpler than realistic catalysts. If the film thickness is in the range of just a few nanometers, the electrical resistance of a nominally electrically insulating material may be so small that the film can be studied with electrochemical methods. Additionally, the flat surface permits to accurately determine the electrochemically active surface area (ECSA), which enables the determination of activity data with small error margins so that the results for different samples can be compared quantitively, thus overcoming a common nuisance in comparative studies of more complex systems.

A few studies of OER in alkaline media employing single-crystal surfaces or epitaxial thin films of Fe, Ni, and Co oxides as anodes have been published in recent years. The earlier work of Bergmann et al.,^5^ Reikowski et al.,^6^ and Wiegmann et al.^7^ have revealed the reversible formation of an oxyhydroxide “skin layer” on Co_3_O_4_(111) surfaces under applied potential. We will also use the term “skin layer” in this publication, meaning the surface layers formed when the samples are introduced into the electrolyte in the presence and absence of applied potential.

Grumelli et al. have shown that the (√2 × √2)R45° surface reconstruction of Fe_3_O_4_(001) is present even at OER conditions, and that it has a profound effect on the reactivity.^8^ Recently, we reported a comparative study of OER on in situ prepared (111)-oriented epitaxial Co_3_O_4_, Co_1+δ_Fe_2−δ_O_4_, and Fe_3_O_4_ films.^9^ Dissolution of small amounts of Fe from the mixed oxide films was found to reduce the surface Fe concentration for the Co_1+δ_Fe_2−δ_O_4_ films during OER and to cause an enhanced reactivity. Fe_3_O_4_ was shown to convert to γ-Fe_2_O_3_.

In recent years, some publications have reported a facet dependence of the OER activity. Müllner et al. studied Fe_3_O_4_(001) and Fe_3_O_4_(110) surfaces, finding that the latter required a lower OER onset potential.^10^ Poulain et al.^11^ studied OER reactivities of NiO films with different preferential orientations deposited on MgO substrates exposing (001), (110), and (111) facets, and found a reactivity trend (110) > (111) > (001). Using electrochemistry and X-ray photoelectron spectroscopy (XPS), this was attributed to differences in the oxyhydroxide phases formed at the respective surfaces during the reaction. For lanthanum nickelate epitaxial thin films grown on oriented strontium titanate single crystals, Füngerlings et al. have studied the facet dependence of the OER reactivity using a combination of electrochemistry, X-ray spectroscopy, and DFT.^12^ They found that the (111) facet is the most active one which they traced back to differences in the formation of the skin layer. Reactivities of electrodeposited Co_0.6_Fe_2.4_O_4_ films with (001), (110), and (111) orientations were compared by Han et al.,^13^ who found the same reactivity trend as observed for NiO by Poulain et al. Recently, Liu et al.^14^ compared single-particle activities of Co_3_O_4_ (001)-terminated nanocubes and spheroids and came to a conclusion contradicting most other studies, i.e, that the (001) surface should be more active than the (111) surface. This was attributed to different abundances of octahedral and tetrahedral cation reaction sites at the two surfaces.

Nonetheless, caution must be applied when comparing with prior work available in the literature. In some studies, the original particle structure (size and shape) might not remain stable during the electrocatalytic process, or if it does, it might be due to the fact that at least some of the particles are not well in contact to the substrate or are exposed to a potential only when they randomly contact the electrode in single-particle experiments. In such cases, the particles might not easily transform from the precatalysts “as-prepared” state to the active state. Moreover, in some studies, calculations are based on the structure of the precatalyst, e.g., Co_3_O_4_, disregarding the well-known experimental fact that an oxyhydroxide skin layer is typically formed on the original oxide surface.

Flat single-crystalline surfaces are ideally suited to study OER facet effects. Here, we compare the OER reactivities of the (001) and (111) surfaces of Co_3_O_4_, CoFe_2_O_4_, and Fe_3_O_4_. In our experimental approach, we prepare and characterize the samples in an ultrahigh-vacuum (UHV) chamber and transfer them without exposure to air to an attached electrochemical cell for electrochemical studies. We employ surface-sensitive techniques such as atomic-resolution scanning tunneling microscopy (STM), X-ray photoelectron spectroscopy (XPS), and low-energy electron diffraction (LEED), to characterize the surface structure, the crystallographic arrangement, and the electronic structure of the surface atoms.

The data reveal that the Co_3_O_4_(001) surface is more reactive than the (111) surface, while the (111) facets are more active for the other film compositions. Furthermore, we find a smaller amount of disordered material after OER on the CoFe_2_O_4_ and Fe_3_O_4_ films than on the Co_3_O_4_ films, indicating that a thinner surface oxyhydroxide layer was formed in the electrolyte. We identify the presence/absence of iron as well as surface structural defects as relevant parameters for these differences. The different arrangement of surface atoms on the (001) and (111) facets is responsible for the observed facet-dependent reactivities, in addition to the effect of surface structural defects. We also found that the initial cobalt hydroxide layer is an active but unstable OER catalyst, making the Co_3_O_4_ performance superior to that of the CoFe and Fe oxides as long as the layer exists. After some time of OER operation, CoFe_2_O_4_(001) with a reduced iron content became the most active catalyst. A Tafel-like evaluation based on LSV scans reveals smaller slopes for the (001) surfaces of all studied films, which is probably mostly related to the different surface structures. Furthermore, the measured “Tafel” slopes are smaller for oxides containing iron, indicating that iron affects the potential-dependent reaction kinetics. Surface defects may play a further role.

## Experimental Section

### Electrochemistry Measurements

The electrochemistry setup and the cleaning procedures are described in the Supporting Information. A central aspect is that the measurements can be done quasi *in situ* without exposure of the samples to air after their preparation under UHV conditions and their characterization with XPS, LEED, and STM.

For all electrochemical data, the given potential scales are *iR*-corrected (the used electrochemical resistances are listed in Table S1) and the potentials are referenced to the reversible hydrogen electrode (RHE). We followed a certain protocol for the electrochemical measurements. After the electrolyte was introduced with the sample at open-circuit voltage (OCV), a potentiostatic electrochemical impedance spectroscopy (PEIS) data set was measured at OCV + 0.04 V, followed by cyclic voltammograms (CVs) comprising an anodic sweep from OCV+0.04 V to the potential where the current density reached 1 mA/cm^2^ followed by a CV cycle between 1 V_RHE_ and the potential corresponding to 1 mA/cm^2^. A PEIS spectrum with the potential at this value was used for *iR* correction. An OER chronoamperometry (CA) scan at the same potential for approximately 2 h followed. The potentials were different for the different oxides (Co_3_O_4_: 1.646, 1.681; CoFe_2_O_4_: 1.708, 1.713; Fe_3_O_4_: 1.714, 1.695 V_RHE_ for (001) and (111) surfaces). Further linear sweep voltammograms (LSVs) were measured after the CA scans: first a cathodic LSV starting from the potential applied during the CA run down to 1 V_RHE_, and then an anodic scan until the current density reached 1 mA/cm^2^. Then, a PEIS spectrum was measured at this condition.

### Thin-Film Preparation

A UHV chamber with a base pressure of 4 × 10^–11^ mbar was used for sample preparation and surface characterization with XPS, LEED, and STM at room temperature, as described in the Supporting Information.

The Fe_3_O_4_(001) and Fe_3_O_4_(111) films were grown on Pt(001) and Pt(111) substrates, respectively, following well-established preparation procedures.^15,16^ Annealing temperatures were restricted to 850 K to prevent dewetting of the films and the consequent exposure of the Pt substrate. The epitaxial Co_3_O_4_(001), Co_3_O_4_(111), and CoFe_2_O_4_(111) thin films were prepared using recipes recently developed by our department.^9,17^

For CoFe_2_O_4_(001) on Pt(001), in the first step a Fe_3_O_4_(001) film was prepared. Co is known to diffuse into Fe_3_O_4_(001) at temperatures of 733 K and higher,^18^ and therefore, the mixed oxide layer was produced by Co deposition onto the prepared Fe_3_O_4_(001) film, followed by annealing at 850 K in UHV for 30 min and then in 5 × 10^–6^ mbar O_2_ for 10 min. Co deposition was repeated until a 1:2 Co/Fe ratio was achieved. XPS with normal angle electron detection was employed to determine the Co/Fe concentration ratio from the Fe and Co 2*p* intensities.

LEED images and some areas of atomic-resolution STM images (Figure S1a–c) of the CoFe_2_O_4_(001) films resemble the (√2 × √2)R45° reconstructed Fe_3_O_4_(001) surface. This structure has been termed the “subsurface cation vacancy (SCV)” reconstruction—in each (√2 × √2)R45° unit cell, two octahedrally coordinated subsurface cations are replaced by a single cation from the tetrahedrally coordinated cation site in the layer above.^19^ This causes the terminating layer to have a distinct appearance in the STM images, with undulations of the atomic rows along [110]. In our CoFe_2_O_4_(001) STM images (Figure S1b,c), we also observe these undulations, but also brighter protrusions scattered over the surface. In fact, the surface looks very similar to that of Fe_3_O_4_(001) with 0.4 ML Co as observed by Bliem et al.^20^ Approximately 20% of the surface area appears somewhat brighter. These areas consist of atoms that nearly always form pairs along a row, and are often aligned with pairs of bright protrusions in the neighboring rows.

De Santis et al. have reported the preparation of CoFe_2_O_4_(001) thin films on Ag(001).^21^ Instead of the (√2 × √2)R45° reconstruction, they observed a (3 × 1) surface reconstruction, which they attributed to segregation of Ag from the substrate to the surface of the film. We were also able to observe such a structure (Figure S1d–f), but without contaminants on the surface. This (3 × 1) surface termination appeared occasionally after deposition of Co and the UHV annealing step in the recipe above, but the reconstruction reverted to (√2 × √2)R45° after the subsequent oxidation step, which may indicate that the (3 × 1) reconstructed surface is somewhat reduced. On the other hand, the (3 × 1) reconstruction was also observed when Fe and Co were deposited simultaneously at 750 K in 5 × 10^–6^ mbar of O_2_. It may be that the (3 × 1) reconstruction is due to an ordering of the protrusions for a slightly reduced state of the oxide.

XPS spectra of CoFe_2_O_4_(001) collected at two different electron detection angles (0 and 70° with respect to the surface normal), see Figure S2, reveal that the surface contains more Fe than the bulk. It may be for this reason that the surface appears to have many similarities with Fe_3_O_4_(001). At the bias voltage used in acquiring the STM images shown in this publication, the surface cations, Co or Fe, dominate the contrast. The presence of Co leads to the brighter areas seen in the STM images of CoFe_2_O_4_(001), which means that the Co atoms cause these areas. Whether the brighter areas are related to Co atoms at the surface or below cannot be concluded from the STM images.

## Results and Discussion

### Surface Characterization

**Figure 1:**
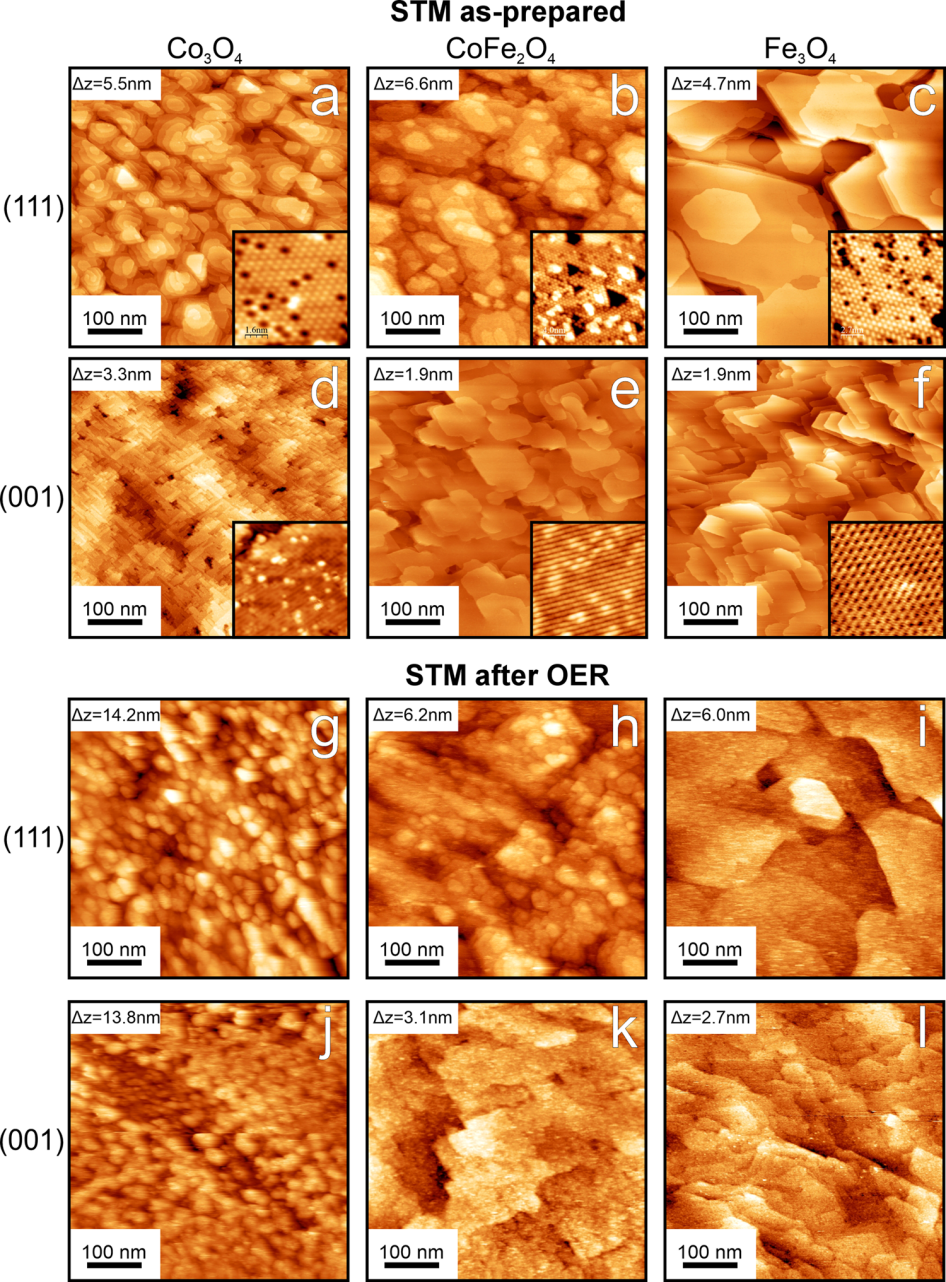
STM images of the Co_3_O_4_, CoFe_2_O_4_, and Fe_3_O_4_ films. Images obtained before (a–f) and after (g–l) a quasi in situ OER run (2 h of CA in 0.1 M KOH solution; the potentials are listed in the Experimental Section). The tunneling conditions are as follows: (a inset, b, c, c inset, e, e inset, h, i, j) sample bias = 2.0 V, current = 0.1 nA, (a, b inset) −2.0 V, 0.1 nA, (d) 1.5 V, 0.1 nA, (f, f inset) 1.5 V, 1 nA, (g) 3.0 V, 0.2 nA. (k, l) 2.0 V, 1 nA, (d inset) 3.0 V, 0.1 nA. The size of all inset images is 8 nm × 8 nm. The Δ*z* given in the large-scale images indicates the total topographical height range of the image. We note that this is different from the RMS surface roughness, which is much smaller, see Table 1.

Large-scale STM images of the oxide films before and after the quasi in situ OER experiments are shown in Figure 1. Surface roughness (RMS) values and scaling factors as determined from STM images with the WSXM software^22^ are listed in Table 1. The scaling factors are the ratios of the STM 3D contour surface areas and the 2D scan areas. From them, the electrochemically active surface areas (ECSAs) were computed by multiplication with the 2D sample area exposed to the electrolyte (0.283 cm^2^). The capacitance of the films at the beginning and the end of a CA under OER conditions was determined via fitting of PEIS data using a model circuit as shown in Figure S3. The specific capacitances in Table 1 are the determined capacitances divided by the ECSAs.

**Table 1:** RMS Roughness, STM Surface Area Scaling Factor, Electrochemically Active Surface Area (ECSA), and Specific Capacitance, *C*_s_, for Co_3_O_4_, CoFe_2_O_4_, and Fe_3_O_4_ Thin-Film Surfaces before and after OER (2 h of CA in 0.1 M KOH Solution; the Potentials Are Listed in the Experimental Section)

	before OER				after OER			
film	RMS roughness (nm)	surface area scaling factor	ECSA (cm^2^)	specific capacitance (mF/cm^2^)	RMS roughness (nm)	surface area scaling factor	ECSA (cm^2^)	specific capacitance (mF/cm^2^)
Co_3_O_4_(001)	0.40	1.01	0.286	0.058	1.20	1.07	0.302	0.049
Co_3_O_4_(111)	1.56	1.04	0.294	0.085	2.21	1.07	0.302	0.086
CoFe_2_O_4_(001)	0.42	1.01	0.286	0.127	0.60	1.03	0.290	0.122
CoFe_2_O_4_(111)	1.09	1.03	0.291	0.067	0.99	1.07	0.302	0.059
Fe_3_O_4_(001)	0.37	1.00	0.284	0.139	0.57	1.02	0.289	0.126
Fe_3_O_4_(111)	0.75	1.01	0.286	0.089	0.70	1.16	0.330	0.062

For as-prepared Co_3_O_4_(001), we see narrow terraces with a width of ca. 10–20 nm, in agreement with the results of Liu et al.^17^ The (111) surfaces of all three oxides have a higher RMS roughness and a larger area scaling factor than the corresponding (001) surfaces, which we attribute to different kinetically controlled growth behaviors. For Fe_3_O_4_(001), the film grows in a layer-by-layer fashion, whereas the Fe_3_O_4_(111)-oriented film grows as islands.^23,24^ This could explain the different RMS roughness values of the (001) and (111) facets of Co_3_O_4_ and CoFe_2_O_4_ films, provided that these growth modes are also operative for these films. The steps on the (111)-oriented films are ∼2.3 times higher than on the (001) films because of the larger distance between equivalent planes along [111].

The as-prepared CoFe_2_O_4_(001) and Fe_3_O_4_(001) surfaces exhibit similarly shaped terraces which are typically 50–100 nm wide. This might be because Fe_3_O_4_(001) was used as the starting point for the preparation of the CoFe_2_O_4_(001) films.

For the Co_3_O_4_ films, the terrace boundaries are not clearly visible after electrochemistry. Some granular features with sizes in the range of 10 nm are observed, leading to a greatly increased RMS roughness and an increased ECSA. These features are attributed to the atomic rearrangement during skin layer formation under OER reaction conditions and their appearance does qualitatively agree with reports that the layer has a rough and open structure.^7,25^ The CoFe_2_O_4_ and Fe_3_O_4_ films retain their general terrace structure for both facets, though the surfaces appear somewhat rougher after OER. The roughness values are not much different before and after OER (see Table 1), indicating less material rearrangement than for the Co_3_O_4_ films.

**Figure 2:**
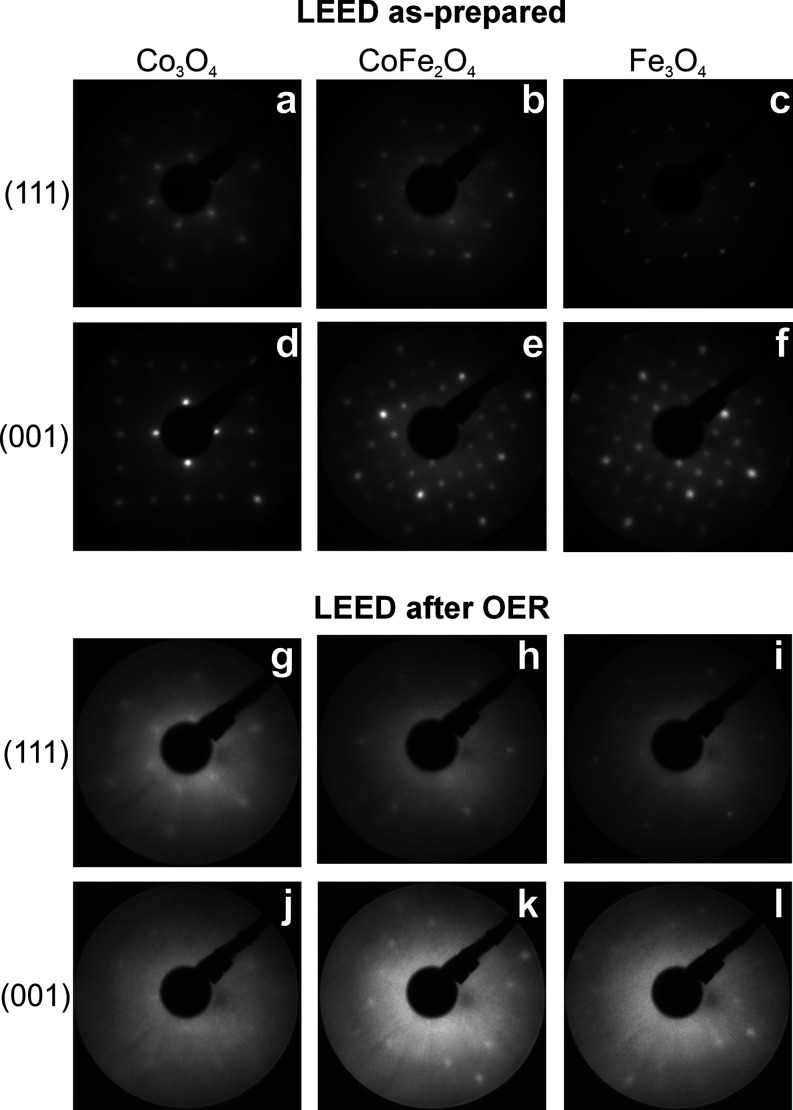
LEED images taken at 160 eV for the Co_3_O_4_, CoFe_2_O_4_, and Fe_3_O_4_ films with (111) (a–c, g–i) and (001) (d–f, j–l) surface orientations. Data recorded (a–f) before and (g–l) after an OER run (2 h of CA in 0.1 M KOH solution; the potentials are listed in the Experimental Section. The brightness of the images has been slightly increased to improve the visibility of weak features.

LEED images of the thin films before and after OER are shown in Figure 2. For all films, a faint spot pattern is visible after OER. The cause for the spot intensity damping and the increased background intensity is the formation of a surface layer also observed in the STM images.

For both, CoFe_2_O_4_(001) and Fe_3_O_4_(001), the (√2 × √2)R45° reconstruction is not visible above the background intensity after the OER experiments, which is at variance with the results of Grumelli et al.^8^ for Fe_3_O_4_(001). It could be that the spots are simply masked by the high background intensity. On the other hand, the Fe_3_O_4_ layers are oxidized under OER reaction conditions as discussed below, and the (√2 × √2)R45° reconstruction might not survive this process.

**Figure 3:**
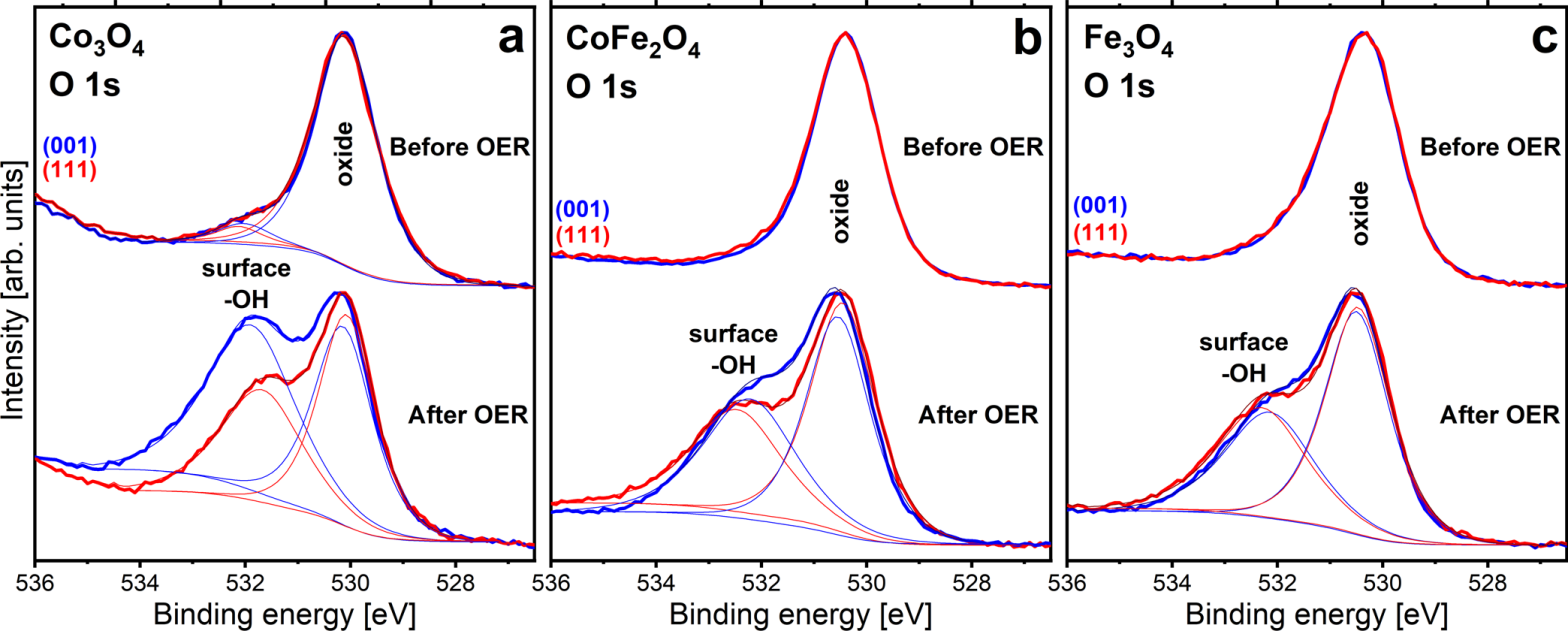
O 1s XPS spectra of the films. Data recorded before and after OER (2 h of CA in 0.1 M KOH solution; the potentials are listed in the Experimental Section) at a detection angle of 70° relative to the surface normal are shown. The thin lines represent fits of the bulk and surface (hydroxyl) groups. The spectra have been normalized to the same peak heights and were shifted along the binding energy axis to align the position of the main peaks for a better comparison. Mg Kα radiation was employed.

**Figure 4:**
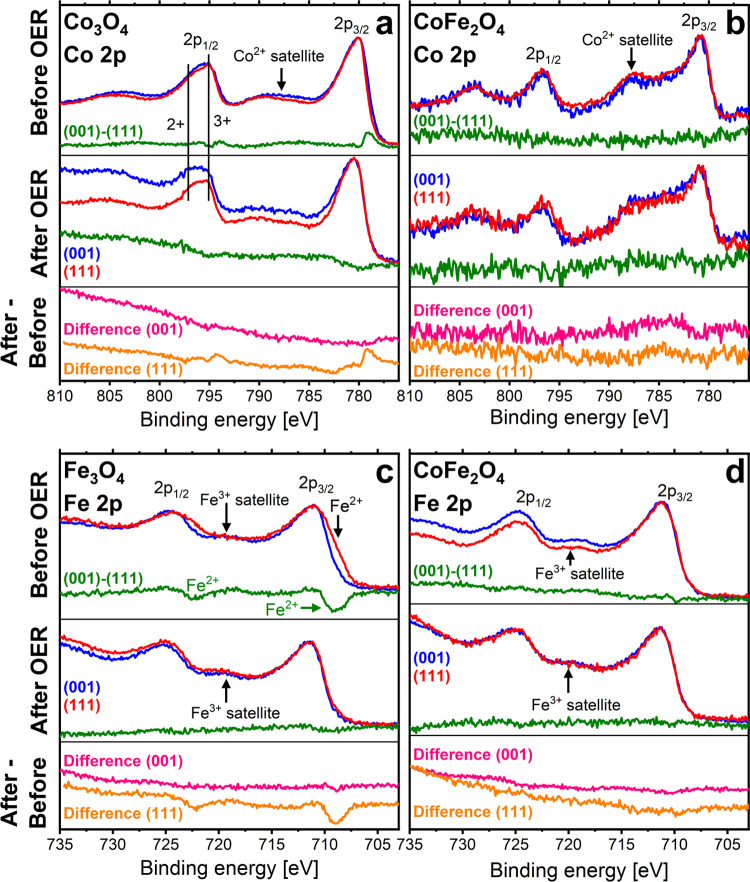
Fe 2p and Co 2p XPS spectra of the films. Data recorded quasi in situ before and after OER (2 h of CA in 0.1 M KOH solution; the potentials are listed in the Experimental Section) at a detection angle of 70° relative to the surface normal are displayed. Several difference spectra are shown for each oxide film: (111) spectrum subtracted from the (001) spectrum (olive lines), “before OER” subtracted from “after OER” for the (001) spectra (pink lines) and for the (111) spectra (orange lines). The spectra have been normalized to the same peak heights and were shifted along the binding energy axis to align the position of the main peaks before computing the difference spectra. Mg Kα radiation was employed.

Figures 3 and 4 show XPS data for the three oxides recorded at an electron detection angle of 70° with respect to the surface normal. The grazing detection angle was chosen to enhance the surface sensitivity. A comparison with normal-emission spectra (0° detection angle) is shown in Figure S4, which displays the spectra without an energy shift.

O 1*s* levels of iron and cobalt oxyhydroxides have been studied with XPS by several authors.^26−34^ O 1*s* binding energies of ca. 529.5–530 and ca. 530.5–531.5 eV were reported for the oxidic oxygen ions and the OH oxygen ions in the oxyhydroxides, respectively. Yang et al. report an O 1*s* binding of 531.2 eV for Co(OH)_2_.^28^ Binding energies of 532 eV and higher were attributed to oxygen ions in adsorbed water or surface hydroxyls. The extra O 1s features seen after OER in Figure 3a–c are all at binding energies above 532 eV, which fits to surface hydroxide or adsorbed water. According to Pourbaix diagrams,^35,36^ CoOOH is not stable under potential-free conditions, and therefore, one would not expect CoOOH-related intensity in the XPS spectra recorded after OER. However, the CoOOH phase may transform so slowly that some of it might still have existed when the XPS spectra were recorded. Thus, the existence of some O 1s intensity stemming from Co(OH)_2_ or OH in CoOOH between the two O 1*s* peaks cannot be excluded in our data, but there are likely not much of these compounds since fitting the O 1s levels in Figure 3 did not require a third peak. Furthermore, the bulk O 1s peaks have similar widths before and after OER, again indicating that there is no bulk hydroxide or oxyhydroxide component with significant intensity. Figure 3a shows that a surface hydroxide very readily forms on the surfaces of the as-prepared Co_3_O_4_ films from the adsorption of trace amounts of H_2_O in the UHV chamber. Thus, it is likely that the Co_3_O_4_ surfaces are already hydroxylated before even coming into contact with the electrolyte.

After OER, the Co^2+^ intensity in the Co_3_O_4_ spectra (Figure 4a) appears somewhat increased relative to Co^3+^, which may be related to the surface hydroxide causing the additional O 1s peak. The Co^2+^ intensity is larger for Co_3_O_4_(001) than for Co_3_O_4_(111), which probably means that there is more hydroxide on the (001) surface than on the (111) surface, as also indicated by the corresponding O 1s spectra (Figure 3a). It also seems that the binding energy of the hydroxyl O 1*s* peak is slightly larger for (001) than for (111), which may result from structural differences in the hydroxides.

Figure 4c reveals that Fe_3_O_4_(001) is oxidized during OER, as seen from the Fe 2p spectra only containing Fe^3+^ after OER (see Figure S4 for less surface-sensitive spectra). The positions of the LEED spots are unchanged, leading us to the conclusion that Fe_3_O_4_(001) is converted to γ-Fe_2_O_3_, similar to the Fe_3_O_4_(111) case.^9^ For as-prepared Fe_3_O_4_, the Fe 2p intensities reveal a higher Fe^2+^ concentration for (111) than for (001), which may be related to different average charge states of the surface atoms. However, after OER, the 2+ intensity is gone due to the transformation to γ-Fe_2_O_3_, and the Fe 2p spectra of the two facets appear similar.

For CoFe_2_O_4_, the oxidation states of the Co and Fe atoms before OER are about the same for both orientations, see Figure 4b,d. In regular CoFe_2_O_4_, the cobalt ions are in a 2+ oxidation state, and therefore, the Co 2p spectrum is very similar to that of CoO.^37^ The iron ions in CoFe_2_O_4_ are nominally in a 3+ oxidation state, which agrees with the Fe 2*p* spectra for both orientations, see Figure 4d. Some additional intensity in the areas around 785 and 800 eV, see Figure 4b, might be attributed to hydroxide.^37^ There is a weak indication of a slightly higher Fe^2+^ concentration at the CoFe_2_O_4_(111) surface, like what was found for the Fe_3_O_4_ films, which would be in line with the enhanced iron concentration at the surface.

**Figure 5:**
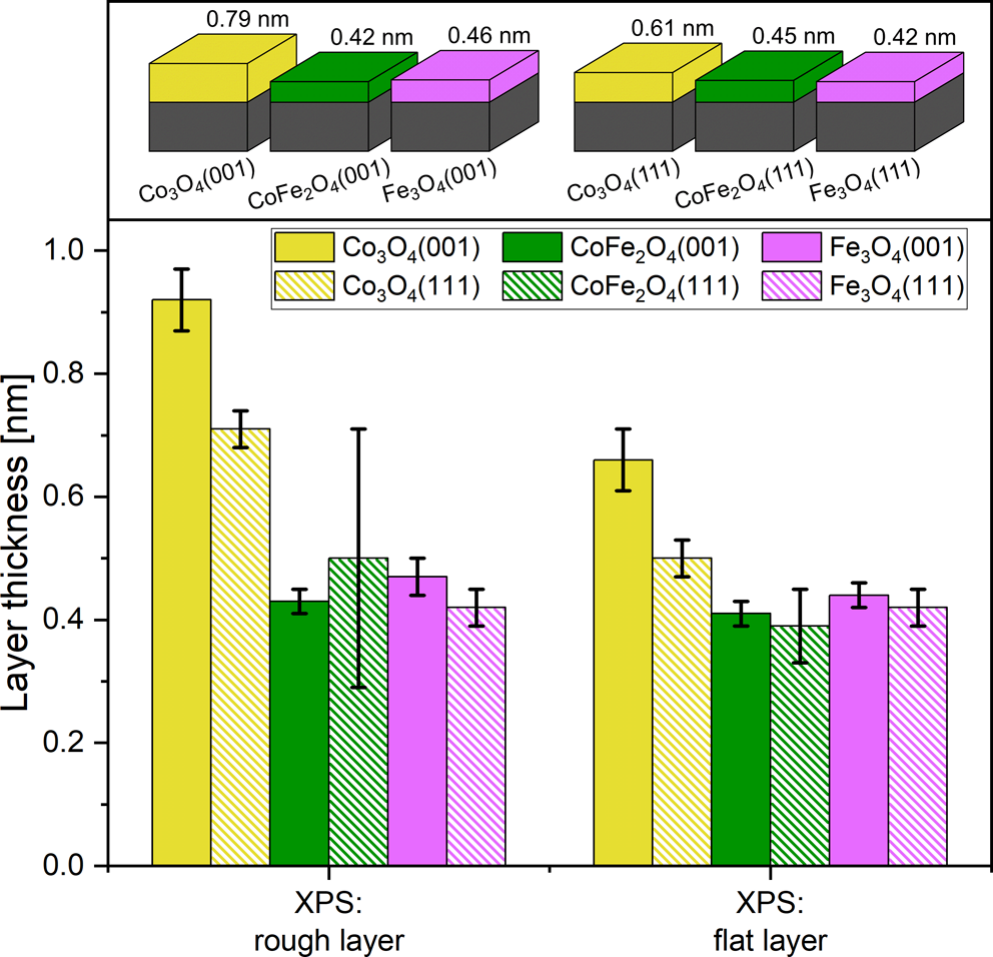
Bottom: skin layer thicknesses computed from XPS O 1s intensity ratios (hydroxyl/bulk) listed in Table S2. The smallest thicknesses compatible with the RMS roughness given in Table 1 and the layer thicknesses computed without consideration of the RMS roughness, just assuming a flat homogeneous layer on a flat substrate are shown. Top: different layer thicknesses; the given numbers are average thicknesses computed from the values in Table S2, which lists the data in numerical form. The error bars are standard deviations derived from two data sets.

The amount of disordered material at the surface is a measure of the amount of oxide material transformed into hydroxide/oxyhydroxide during OER. Knowing the amount and especially the effect of iron on it might aid a better understanding of the OER reaction on the studied oxides. We followed two approaches to determine the thickness: one approach is based on the quantitative evaluation of the surface hydroxide peak intensity relative to that of the oxide bulk peak in the O 1s spectra. This yields the amount of material giving rise to the extra O 1s peak. However, as discussed above, the additional O 1s peak does not contain contributions from bulk hydroxide and oxyhydroxide. Furthermore, it just refers to the layer at the surface after OER, but not to the layer present at the surface during OER. Therefore, modeling the damping of the LEED spot intensities by the skin layer was employed as another approach to estimate the surface layer thickness. Here, we assume that the skin layer formed during OER left behind a disordered layer on the surface instead of transforming back into the crystalline oxide. This layer would weaken the LEED spot intensity. In this scenario, the LEED-spot-intensity approach determines the amount of material transformed into (oxy)hydroxide during OER and not just its amount after OER. For both approaches, rather simple equations are available to determine the skin layer thickness if this layer is flat and homogeneous. However, the surfaces are not really flat, see Table 1, and therefore, we used a simple approach to include the effect of the roughness in an approximate way. Details are discussed in the Supporting Information (Figure S5 and the accompanying text). We note that especially the LEED-spot-damping approach has a significant margin of uncertainty, which is why the determined values should be taken as estimates. Figure 5 presents the XPS-derived results graphically. The skin layer thicknesses derived from the LEED spot damping data (Figure S6) are not massively different from those derived with the XPS-based approach, which leads us to conclude, that the skin layers are not much thicker during the OER run than after it and that there is probably not much “bulk” (oxy)hydroxide in the skin layers after OER, if any at all. Table S2 lists the data plotted in Figure 5 and Figure S6 in numerical form. For both, the XPS- and the LEED-based approach, two values for the skin layer thickness are given—one for a flat layer and one for a rough layer. The latter is the smallest thickness compatible with the given roughness (see Table 1) under the conditions listed in the Supporting Information. As expected, the rough-layer thickness is a bit larger. Table S2 reveals that the layer thickness is below 1 nm in all cases. We note that the accuracy of the thickness derived from LEED spot damping (Figure S6) for Fe_3_O_4_ may suffer from the oxidation of the oxide during OER, which affects the LEED spot intensities.

Wiegmann et al. investigated skin layers formed under applied potential in 0.1 M NaOH on epitaxial Co_3_O_4_(111) layers on Ir(001) prepared under UHV conditions.^7^ These layers may be qualitatively similar to those employed in this study. They concluded from *operando* surface X-ray diffraction (SXRD) data that the thickness of the layer formed on an UHV-produced oxide film was “insignificant”,^7^ which means that it was so thin that they could not clearly detect it, while clearly detectable skin layers were found on epitaxial electrodeposited films. The difference was discussed in terms of the regular surface structure of UHV-deposited films, but it may be assumed that a higher defect density at the surface of the electrodeposited films does also play a role, indicating that investigating the role of defects could be an important topic. Our UHV-grown films were annealed to reduce the defect density and the surface structural quality was checked with surface-sensitive methods such as STM and LEED. The surfaces produced in this way are still not defect-free, but the electrodeposited films were much less optimized with respect to their structure, which did probably lead to a notably higher defect density which might affect the skin layer growth.

According to Reikowski et al.,^6^ the skin layers should transform back into the original crystalline state when the potential is removed, which would affect the accuracy of the LEED-based results. On the other hand, the LEED patterns obtained after OER (Figure 2g–l) clearly indicate the presence of a disordered phase after OER so that the back-transformation previously described in the literature cannot be complete. Also, the STM images recorded after OER (Figure 1g–l) reveal the formation of surface structures that are likely not crystalline. We cannot exclude that there is also a part of the skin layer that back-transforms into crystalline oxide. However, as mentioned before, the skin layers on UHV-grown films are so thin that Wiegmann et al. could not detect them in an operando experiment with SXRD,^7^ which sets a limit to the amount of skin-layer material back-transformed into crystalline oxide. We also mention that the skin-layer material undergoes several phase transitions from the state before OER to the state after OER (tentative and simplified: crystalline oxide → hydroxide → oxyhydroxide → hydroxide → oxide), which would probably impact the crystallinity.

The facet’s crystalline orientation and the presence/absence of iron will surely affect the growth of the skin layers. As already discussed, there is quite likely also a promoting influence of structural defects. The STM data (Figure 1) do not really permit to establish convincing correlations between the surface structure and the defect density, but they give the impression that the density of steps and other line boundaries on the cobalt oxide precatalyst surfaces might be somewhat larger than on the other films (except perhaps CoFe_2_O_4_(111)), which would support the hypothesis that the defect density matters since the thickest skin layers were found on the cobalt oxide films. Point defects may also play a role, but a reasonable density is not just found on the cobalt oxide layers but also on CoFe_2_O_4_(111) and Fe_3_O_4_(111). Of course, the effect of defects on the skin layer growth would likely be different for different facets and compositions.

For the cobalt oxide films, it might be that the granular structure of the skin layers promotes further skin layer growth. The granularity might permit that the electrolyte can still interact with the oxide when there is already a skin layer, thus promoting its further growth. The skin layers are smoother on the films which contain iron. Thus, the role of iron could also be that it leads to smoother and more closed layers, protecting the underlying oxide better from the interaction with the electrolyte. The O 1s XPS data exhibit larger hydroxyl peaks for the cobalt oxide films even if the samples were just exposed to UHV conditions (top row of spectra in Figure 3), indicating that the absence of iron makes the layers more sensitive to hydroxylation, which is somewhat in line with the observation of thicker skin layers after OER on the cobalt oxides.

For Co_3_O_4_, there is clear skin layer thickness difference between the (001) and (111) facets in all data sets, with a larger thickness for the (001) facet, while for the other films, CoFe_2_O_4_ and Fe_3_O_4_, there is no clear facet dependence. It may play a role that the density of surface metal centers is larger on the (001) facets than on the (111) facets; it might also be that the oxyhydroxide skin layer just grows quicker on Co_3_O_4_(001) for kinetic reasons. Another explanation is that a high defect density on Co_3_O_4_(001) is responsible. The STM images of Co_3_O_4_(001) (Figure 1) reveal structures at much smaller distances than on the other oxides so that it might well be that a high surface defect density is actually responsible for the comparably large thickness of the skin layer on Co_3_O_4_(001).

We note that the skin layers especially on the Fe_3_O_4_ and CoFe_2_O_4_ films are so thin, that there may be just a monolayer-type skin under OER reaction conditions, which would simplify computational modeling approaches that we hope the present work will inspire.

All oxide layers have been subjected to an electrochemical characterization. There are publications that report a direct effect of the metallic substrate material on the catalytic OER activity (see for instance Yeo and Bell^38^ and Fester et al.^39^). We assume that this does not apply to the films studied here thanks to the thickness and non-negligible defect concentration. A short discussion of this topic is provided in the Supporting Information.

**Figure 6:**
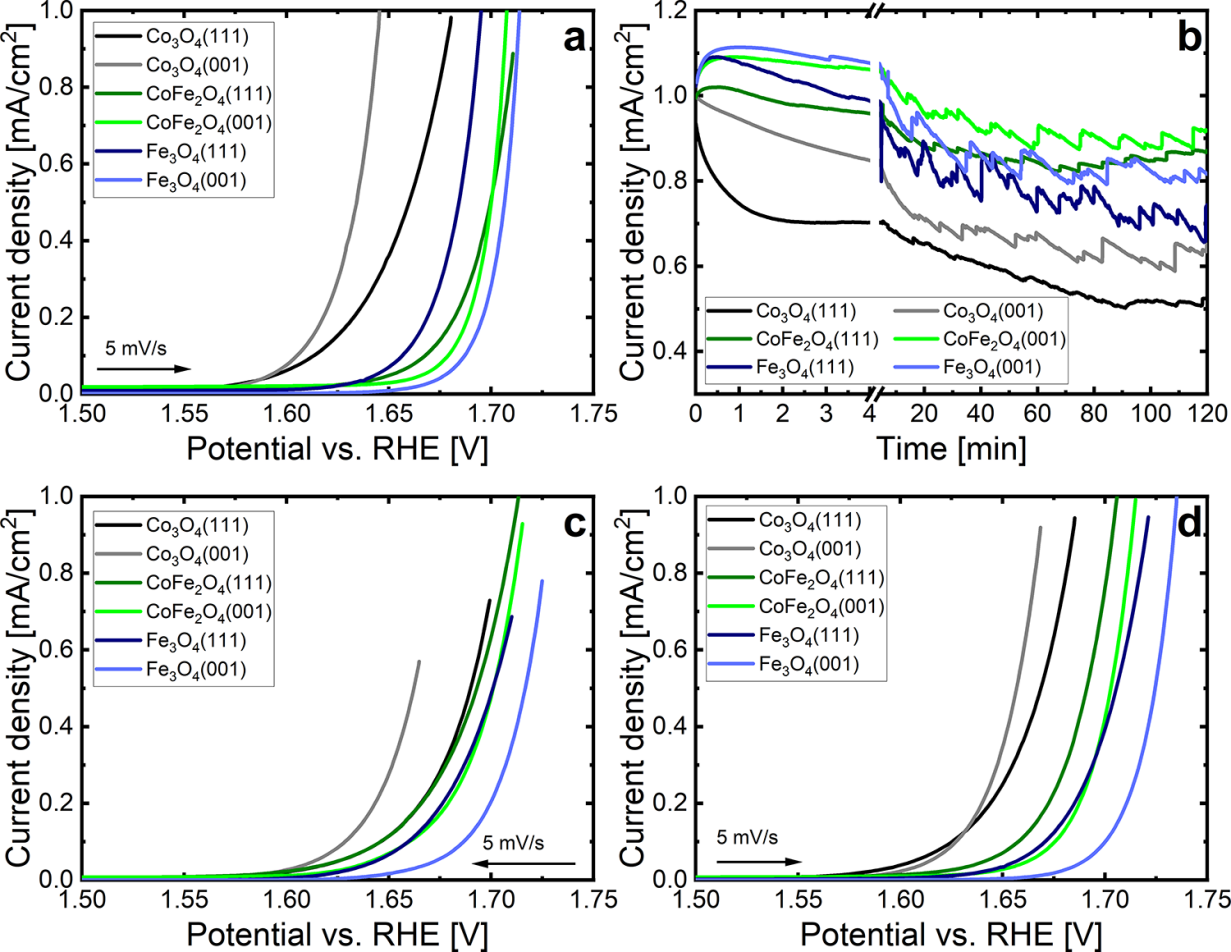
Electrochemical data for the thin oxide films. (a) Initial anodic LSV from OCV to OER conditions, (b) 2 h CA measurement at OER conditions, (c) cathodic and (d) anodic LSVs measured immediately after the CA, first the cathodic scan and then the anodic one. The CA scans were performed with the potential where the current density reached 1 mA/cm^2^ as listed in the Experimental Section.

Figure 6a shows LSVs in the region of the OER onset for the as-prepared films. The measurements started slightly above OCV and went up to the potential where the OER reaction was active with a current density of 1 mA/cm^2^. Following this initial LSV, the potential was swept down to 1 V_RHE_, then back up to the potential where the current density reached 1 mA/cm^2^. The potential was held for at least 2 h, and chronoamperometry (CA) data were measured to reveal the evolution of the current, see Figure 6b. Following the CA measurements, a cathodic LSV down to 1 V_RHE_ was performed, followed by an anodic LSV scan to OER conditions. These are shown in Figure 6c,d. In Figure 6c, the start potential of the LSV curves for each film is equal to the CA potential.

**Figure 7:**
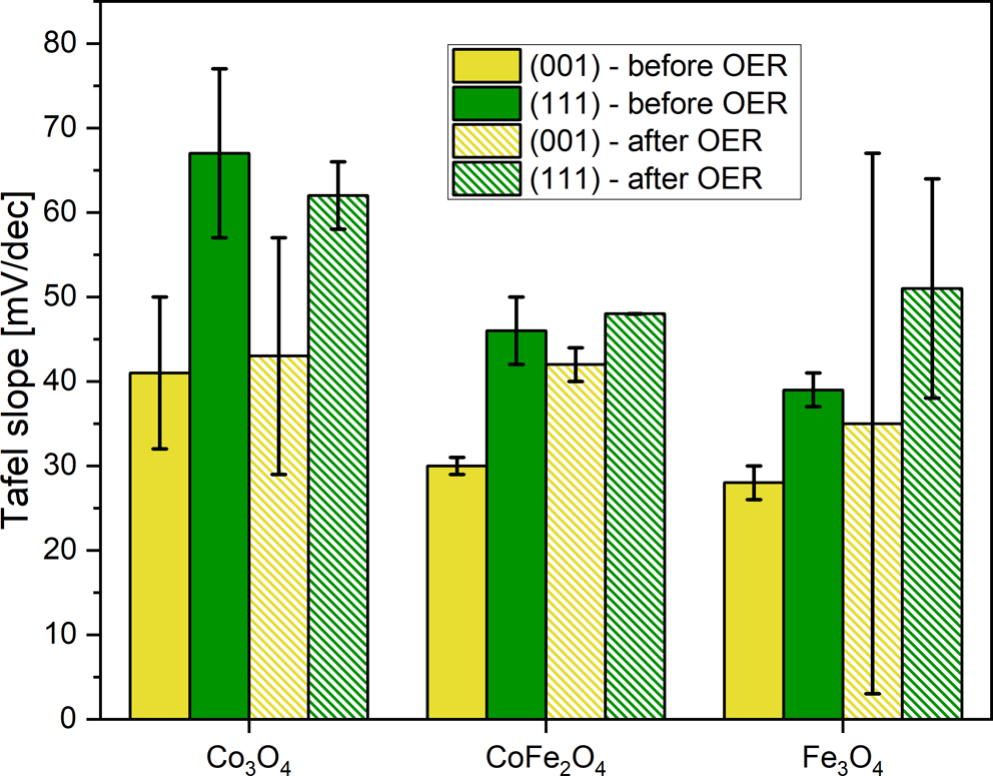
“Tafel” slopes obtained from anodic scans before and after 2 h of OER. The slopes are derived from the LSV data shown in Figure 6a,d. The error bars were derived from two data sets and the slopes are averages.

The LSV curves in Figure 6 exhibit differently steep current increases when the potentials are scanned. To quantitatively access this, we have plotted the LSVs in Figure 6a,d in a Tafel-like style (Figure S7). Figure S7a shows data for the fresh catalysts (LSVs from Figure 6a) and Figure S7b for the same catalysts after 2 h of OER reaction (LSVs from Figure 6d). These graphs are not fully qualified Tafel plots, where for every point the catalyst state is stable, but just Tafel-like graphs. We note that the slow scan speed of 5 mV/s gives the system time to adapt to some extent to the changing potential so that the difference to real Tafel plots may be limited. In the following, we present a qualitative comparative discussion where the errors might cancel to some extent due to the comparative nature. We have used the largely linear parts above 0.8 mA/cm^2^ of the curves in Figure S7 to determine the “Tafel” slopes which are displayed graphically in Figure 7 (Table S3 lists them numerically). The reasonable linearity of the “Tafel” curves in Figure S7 indicates that the reaction mechanism does not change significantly in the plotted range.

Tafel slopes for Co_*x*_Fe_3–*x*_O_4_ nanoparticles with different compositions (0.25 ≤ *x* ≤ 3) were published by Saddeler et al.,^40^ matching the numbers found for the thin films reasonably well, with values for Co_3_O_4_ NPs of ∼61 mV/dec (thin films: 41–67 mV/dec, see Table S3) and ∼43 mV/dec for CoFe_2_O_4_ (thin films: 30–48 mV/dec, see Table S3). The slopes for the thin Fe_3_O_4_ films must be viewed with some suspicion since this oxide transforms to γ-Fe_2_O_3_ during OER. Nevertheless, Gao et al.^41^ reported a number in the range of 50 mV/dec for Fe_3_O_4,_ which is at least not too far off from the numbers reported here for the epitaxial films (28–51 mV/dec, see Table S3). This level of agreement gives some trust that the “Tafel” slopes determined here are reasonable. Despite the relatively large error bars, one may conclude that the slopes for the (001) facets are smaller than those for the (111) facets. The (001) facets exhibit 4-fold and 5-fold coordinated metal ions, while the (111) facets are terminated with 3-fold coordinated metal ions, at least on UHV-grown samples (this has not been proven for the cobalt ferrite surfaces, but it may also be the case here). On the other hand, the Co_3_O_4_(001) surface exhibits a more open crystal structure, which allows for easier metal ion migration of the tetrahedrally coordinated Co ions compared to the Co_3_O_4_(111) termination. A more facile ion migration would help in the formation of the CoO_*x*_(OH)_*y*_ skin layer in which the metal ions are predominantly octahedrally coordinated. Furthermore, the Co_3_O_4_(001) precatalysts contain a higher density of low coordination oxygen sites which have been linked to enhanced OER kinetics.^5^ Thus, we expect a combination of these effects to determine the lower “Tafel” slope for the (001) facets. Interestingly, although the Fe_3_O_4_ films have the largest overpotential, they also have the smallest “Tafel” slopes. As discussed, the Fe_3_O_4_ films are oxidized to γ-Fe_2_O_3_ under OER reaction conditions, which might affect the slopes. However, the CoFe_2_O_4_ films, which are largely Fe-terminated, but are not oxidized under reaction conditions, also have consistently smaller slopes than those of the cobalt oxide films, suggesting that the small Fe_3_O_4_ “Tafel” slopes are not exclusively related to the iron oxidation process taking place during OER. Further information that may be derived from the “Tafel” slopes in Figure 7 is that the slopes for the films containing iron seem to be somewhat smaller than those for the cobalt oxide films, indicating that the addition of iron has a favorable effect on the potential-dependent reaction kinetics.

The overpotentials at 1 mA/cm^2^, i.e., the potentials relative to the H_2_O/O_2_ equilibrium potential of 1.23 V_RHE_ at the given current, are larger than 400 mV in all LSV curves in Figure 6. This shows that the reactivities of the epitaxial thin films lag behind those of optimized catalysts. Overpotentials at the even higher current density of 10 mA/cm^2^ of less than 200 mV or even below 100 mV were reported for reactive systems.^42^ Many systems have been studied in recent years and therefore further studies of catalyst systems with lower overpotentials than those of our model systems may be easily found in the literature. High overpotentials are typical for model systems that rely on structural simplicity, targeting a fundamental understanding of the catalytic processes and the relevant parameters governing the catalytic performance. Optimizing the catalytic performance may come as a follow-up step where systematic modifications of the model systems are studied, targeting the improvement of the catalyst’s performance.

**Figure 8:**
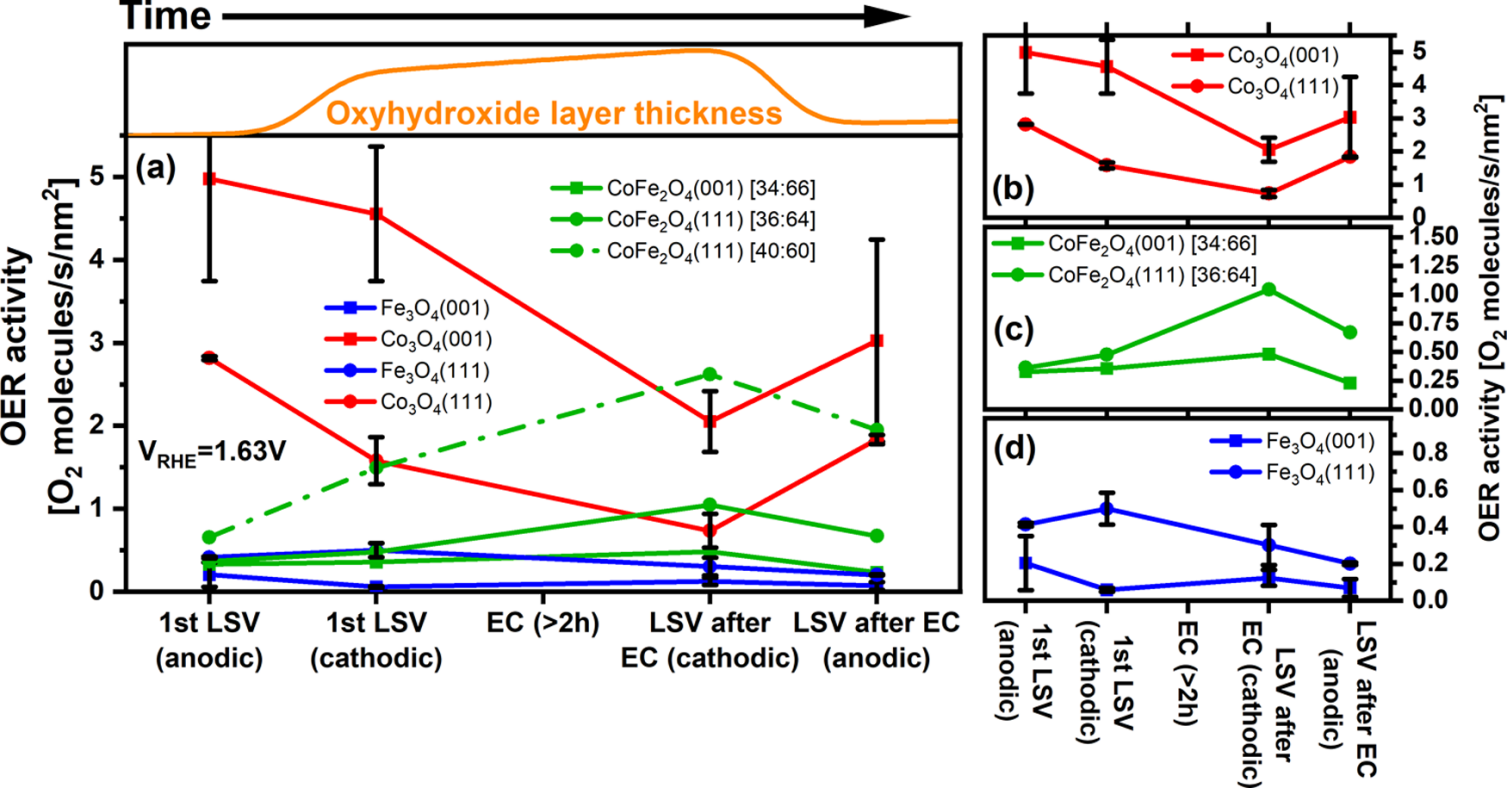
OER activity of the thin films at various stages of the experiment. (a) Comparison of the activity data of all studied layers, (b–d) comparison of the (001) and (111) facet activities for the three oxides using adapted ordinate scales. The numbers in square brackets given for the cobalt ferrites films are the Co:Fe surface concentration ratios obtained from XPS data. To illustrate the effect of the iron concentration, in (a), a curve for a somewhat deviating concentration ratio, 40:60, is shown. The oxyhydroxide layer thickness plot on top of (a) is a qualitative estimate based on a report that the thickness increases with increasing potential and decreases when the potential is lowered.^6^ The error bars are standard deviations derived from two or more measurements. For CoFe_2_O_4_ films, error bars are not available since the prepared oxides had always slightly different Co:Fe compositions, and therefore the data were not averaged in view of the strong impact of the composition on the OER reactivity.

The evolution of the activity of the catalysts and its facet dependence are most clearly seen in Figure 8, which shows the number of O_2_ molecules produced per second × nm^2^ during the LSVs at V_RHE_ = 1.63 V, 0.4 V above the OER equilibrium potential. The first two abscissae points represent the first anodic and cathodic LSV scans after the sample was introduced into the electrolyte. The third point, “LSV after EC (cathodic)”, probably represents best the O_2_ production activity under stationary conditions, since these values are extracted only seconds after the end of the 2 h CA runs. The last point represents the subsequent anodic scan. Table S4 lists the data in numeric form.

The Co_3_O_4_ layers exhibit the highest currents in the initial anodic and cathodic LSV scans, see Figure 8. In this early stage, the catalyst is still changing toward a stable state. Part of the current may be consumed for the transformation (mostly oxyhydroxide layer formation) and only the rest would be available for O_2_ production. Therefore, quantitative statements about the OER reactivity are not straightforward. However, the electrochemical currents are also the largest for the first cathodic scan, which is a hint that there is a significant OER contribution.

A more stable state is represented by the point “LSV after EC (cathodic)” in Figure 8, since here the OER reaction has been running for 2 h. At this point, the CoFe_2_O_4_(111) [40:60] film with a cobalt-to-iron ratio of 40:60 has the best activity while the CoFe_2_O_4_(111) [36:64] layers lag significantly behind, demonstrating the critical effect of the initial iron concentration. We note that the given concentrations have been derived from XPS spectra taken at normal emission, which are not very surface-sensitive and therefore the concentrations at the very surface may be somewhat different, and we also note that the surface concentrations may change during the OER reaction due to iron leaching as found before for CoFe_2_O_4_(111).^9^

For both Co_3_O_4_ layers, the activity had decreased after the OER run with respect to the initial two scans. In the cathodic scan after OER, the potential is ramped down to 1 V_RHE_, i.e., to a potential where the surface layer formed under OER reaction conditions is not stable anymore. In the following anodic scan, the electrochemical current is larger for both Co_3_O_4_ facets, suggesting that the layer formed at sub-OER potential, probably a hydroxide layer, is more OER-active than the layer produced under OER conditions, which likely consists of a significant part of an oxyhydroxide. A similar observation was also reported by Davis et al.^9^ We note that part of the electrochemical current may be related to skin layer rearrangements/charging of the double-layer capacitor instead of O_2_ production but we assume that this contribution is rather small given the limited thickness of the skin layer and the low scan rate (5 mV/s).

The (001) termination is the more active of the two studied Co_3_O_4_ terminations. A similar result has been reported by Liu et al.,^14^ who traced this back to different reactivities of cations at the Co_3_O_4_(001) and (111) oxide surfaces. A clear difference between the skin layers on Co_3_O_4_(001) and Co_3_O_4_(111) is that the one on the (001) facet is thicker, see Figure 5. A relationship between the skin layer thickness and the reactivity has been reported before based on experimental data, and it was indicated that the OER reaction does not just occur at the very surface but that the whole skin layer is involved. This was tentatively attributed to the presence of pores in the oxyhydroxide layer, its roughness, and a defect-related enhanced oxygen mobility.^7^ Thus, the larger reactivity of the (001) facet may at least in part be a consequence of the thicker skin layer. In addition, the structures of the skin layers on the two oxides are likely somewhat different due to the different underlying oxide facets. For the thin oxyhydroxide layers discussed here, it is quite likely that they are different from known oxyhydroxide phases since the interaction with the substrate may enforce new, substrate-dependent structures, which will also affect the OER reactivity. It should be mentioned that prior theoretical work looking at the facet effect has been often based on comparing the structures of Co_3_O_4_ with different surface terminations, instead of comparing the reactivity of two oxyhydroxide skin layers with different structures.

The CA current density decreases immediately after the start for both Co_3_O_4_ films, see Figure 6b. For the (111) film, this decrease is rapid during the initial 2 min, but for the (001) film a much more gradual decrease is observed. We speculate that this might be due to a slower buildup of the CoOOH skin layer on the (001) facet. One reason for this may be the larger amount of CoOOH formed on the (001) facet, see Figure 5, which may simply take a bit longer to build up. The initial OER activity of Co_3_O_4_(001) surpasses that of all other oxide films at all points in Figure 8, before and after the CA runs, which underlines the high OER reactivity of the pre-existent cobalt hydroxide layer.

The results do largely agree with those of Buchner et al., who compared several Co/Fe oxides thin films.^43^ The reported Co_3_O_4_(111), Fe_3_O_4_(001), and CoFe_2_O_4_ overpotentials are essentially in agreement with the reactivities reported here in that they follow the same order between the oxide films.

As discussed above, iron appears to hinder the hydroxide/oxyhydroxide layer growth, see Figure 5, which would have a negative impact on the OER reactivity, considering just the dependence of the reactivity on the oxyhydroxide layer thickness postulated previously.^7^ However, the CoFe_2_O_4_(111) [40:60] film is the most active under reaction conditions despite the smaller skin-layer thickness compared to Co_3_O_4_(001). This means, that the enhancing effect of iron on the OER reactivity is not related to an increased skin-layer thickness in the given case. Here, additional aspects such as variations in the thermodynamics of charge accumulation or redox electrochemistry, which we unfortunately could not resolve in our electrochemical experiments, can lead to enhanced activity and thus counterbalance the effect of the reduced oxyhydroxide layer thickness for the Fe-containing oxide films. It has been shown clearly for Ni-based electrocatalysts that the redox electrochemistry changes in the presence of iron while the effect is weaker for Co-based electrocatalysts.^44,45^

The skin layers formed on CoFe_2_O_4_ and Fe_3_O_4_ films at low potential, probably hydroxide, seem to be less reactive than the oxyhydroxide-containing layers formed at higher potential under OER reaction conditions, which is opposite to the Co_3_O_4_ case. Also, the facet dependence is inverted: the Fe_3_O_4_ and CoFe_2_O_4_ (111) facets are more reactive than the (001) facets at all points (see Figure 8b–d). We attribute both effects to the presence of iron.

The films with Fe at the surface, i.e., the Fe_3_O_4_ and CoFe_2_O_4_ films, all exhibit an initial increase of the current density during the first couple of minutes of the CA, see Figure 6b, while the current density for the Co_3_O_4_ films decreases immediately. This arises from the different reactivity ratios of the hydroxide and oxyhydroxide layers on Fe_3_O_4_ and CoFe_2_O_4_ (which are largely iron terminated) on the one side and Co_3_O_4_ on the other side. For the CoFe_2_O_4_ films, the decrease is less pronounced, and after ∼1 h the current density is stabilized, which we attribute to a decreasing iron concentration due to iron dissolution shown previously for CoFe_2_O_4_(111).^9^

## Conclusions

In summary, we present a comparative study of epitaxial Co_3_O_4_, Fe_3_O_4_, and CoFe_2_O_4_ thin films with two different crystallographic surface terminations, (001) and (111). The films have well-known ECSAs and were characterized by sensitive-surface science methods (LEED, XPS, and STM). Our aim was to perform experiments that permit to compare the activities and other data quantitatively, targeting the effect of iron on the OER reactivity by using epitaxial samples with well-defined ECSA, composition, and structure. Such an effort has not been undertaken previously, at least not to this extent. As discussed in the text, it appears that there is probably a strong effect of structural inhomogeneities on the electrochemical activity and therefore we think that such a strict approach is required to derive reliable conclusions. Still, it appears that there is an effect of surface defects even for these rather well-defined layers. This is a topic that might be investigated in forthcoming studies. Some of the topics discussed here have also been studied by other authors who came to similar conclusions, but we feel that the stricter approach chosen here puts the results on a more solid experimental basis.

We found several potential descriptors governing the facet and composition dependence of the (oxy)hydroxide skin layer growth and the OER reactivity.

(1) The thickness of the skin layers is below 1 nm for all studied layers under the given experimental conditions. We find that the thickness depends on the composition (Co/Fe) and the facet orientation. Structural defects are discussed as additional relevant parameters. The presence of iron in the films appears to lead to thinner skin layers.
(2) The OER reactivities are facet-dependent. This may be traced back to the different facet structures, with the (001) facets exposing four and 5-fold-coordinated metal ions while the (111) facets expose only 3-fold-coordinated ones. Our work nicely features that the underlying oxide surface structures will affect the structures of the thin skin layers which are directly involved in the reaction. For Co_3_O_4_, the (001) facet is clearly more reactive, while for the other oxides, the (111) facet is more active. Also here, surface structural defects may play a role.
(3) Iron inverts the ratio of the reactivities of the skin layers formed at sub-OER potentials and those formed under OER reaction conditions: for Fe_3_O_4_ and FeCo_2_O_4_, the oxyhydroxide layer formed under OER conditions is more active, while for Co_3_O_4_ the hydroxide layer initially present in the as-prepared samples or formed at sub-OER potential is more active.
(4) “Tafel” plots indicate a systematic dependence of the slope on the facet orientation: the slopes for the (001) facets are smaller than those for the (111) facets, which points toward a more favorable potential-dependent reaction kinetics on (001) facets. Moreover, the “Tafel” slopes are systematically smaller for oxides containing iron than for the pure cobalt oxide films, indicating that iron improves the potential-dependent reaction kinetics.
(5) An interesting effect is the high OER reactivity of Co hydroxide layers. Though not of much practical relevance due to the instability of this hydroxide under OER reaction conditions, this may be a topic for theoretical studies to improve the understanding of OER processes on cobalt oxides.

It seems that structural defects may critically affect the reactivities. This demands further studies where this topic is investigated in some detail.

## Supporting Information Available

LEED and STM data of CoFe_2_O_4_(001); iron concentrations derived from XPS data with different surface sensitivities; double Randles circuit (evaluation of PEIS data); XPS data for two different detection angles; procedure for the computation of the layer thickness; OER activity data; computed layer thicknesses; “Tafel” slopes; experimental details (electrochemical cell, UHV chamber); and discussion of the effect of the metal substrate on the electrochemical results (PDF)
