# Supplementary material for: Facet Dependence of the Oxygen Evolution Reaction on Co3O4, CoFe2O4, and Fe3O4 Epitaxial Film Electrocatalysts
Source: J Am Chem Soc. 2024 May 8;146(20):13770–82. doi: 10.1021/jacs.3c13595 (PMC11117179; doi:10.1021/jacs.3c13595)
Supplement: Supplementary file 1 — ja3c13595_si_001.pdf [file ja3c13595_si_001.pdf]

Facet Dependence of the Oxygen Evolution Reaction on Co_3O_4, $CoFe_2O_4$, and Fe_3O_4 epitaxial film electrocatalysts

Supplementary information

Earl Matthew Davis, Arno Bergmann, Helmut Kuhlenbeck*, Beatriz Roldan Cuenya*

Department of Interface Science, Fritz Haber Institute of the Max Planck Society, 14195 Berlin, Germany

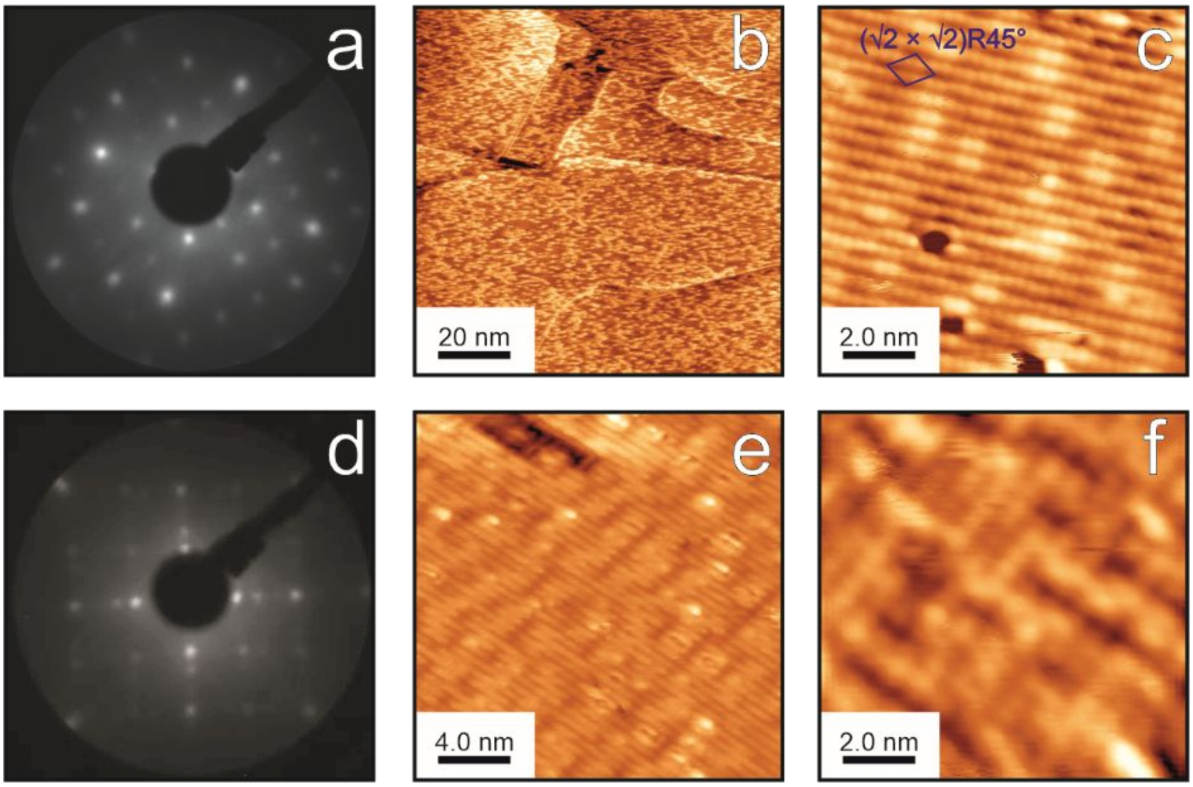

Figure S1 *– $CoFe_2O_4$(001) LEED images of a) the (√2 × √2)R45° surface termination, and d) the (3 × 1) surface termination, both acquired at 130 eV. STM images of $CoFe_2O_4$(001) acquired from b,c) the (√2 × √2)R45° surface termination, and e,f) the (3 × 1) surface termination. Tunnelling conditions: Sample bias = +1.5 V, tunnelling current = 0.10 nA (0.05 nA for (f)).*

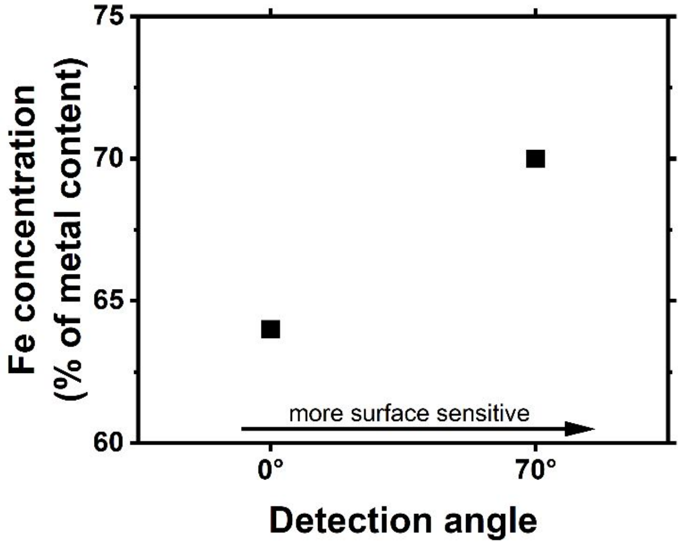

Figure S2 *– Fe concentration, as a percentage of total metal concentration, for a freshly prepared $CoFe_2O_4$(001) film. Data acquired at a detection angle of 70° are more surface sensitive by about a factor of 3. The data shown here suggest that the concentration of Fe at the surface of a freshly prepared film is higher than in the bulk of the film. The IMFP (inelastic mean free path length) of the Fe2p photoelectrons is ~1.65 nm as computed with the Quases IMFP-TPP2M program.*[1] *For normal electron detection (0°), 63% of the photoelectrons originate from a surface layer with this thickness (~86% from a layer with twice the thickness). The corresponding thicknesses are smaller by a factor of ~3 [1/cos(70°)] for photoelectrons leaving the surface at an exit angle of 70°. i.e., 0.48 and 0.96 nm for 63% and 86% of the photoelectrons, respectively.*

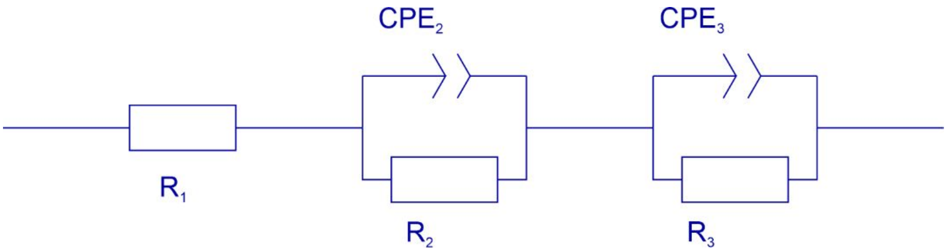

Figure S3 *– The double Randles circuit used to model the impedance spectra, comprising a serial resistor, R_1, and two Randles circuits R_2/CPE_2 and R_3/CPE_3. CPE=constant phase element. R=resistor.*

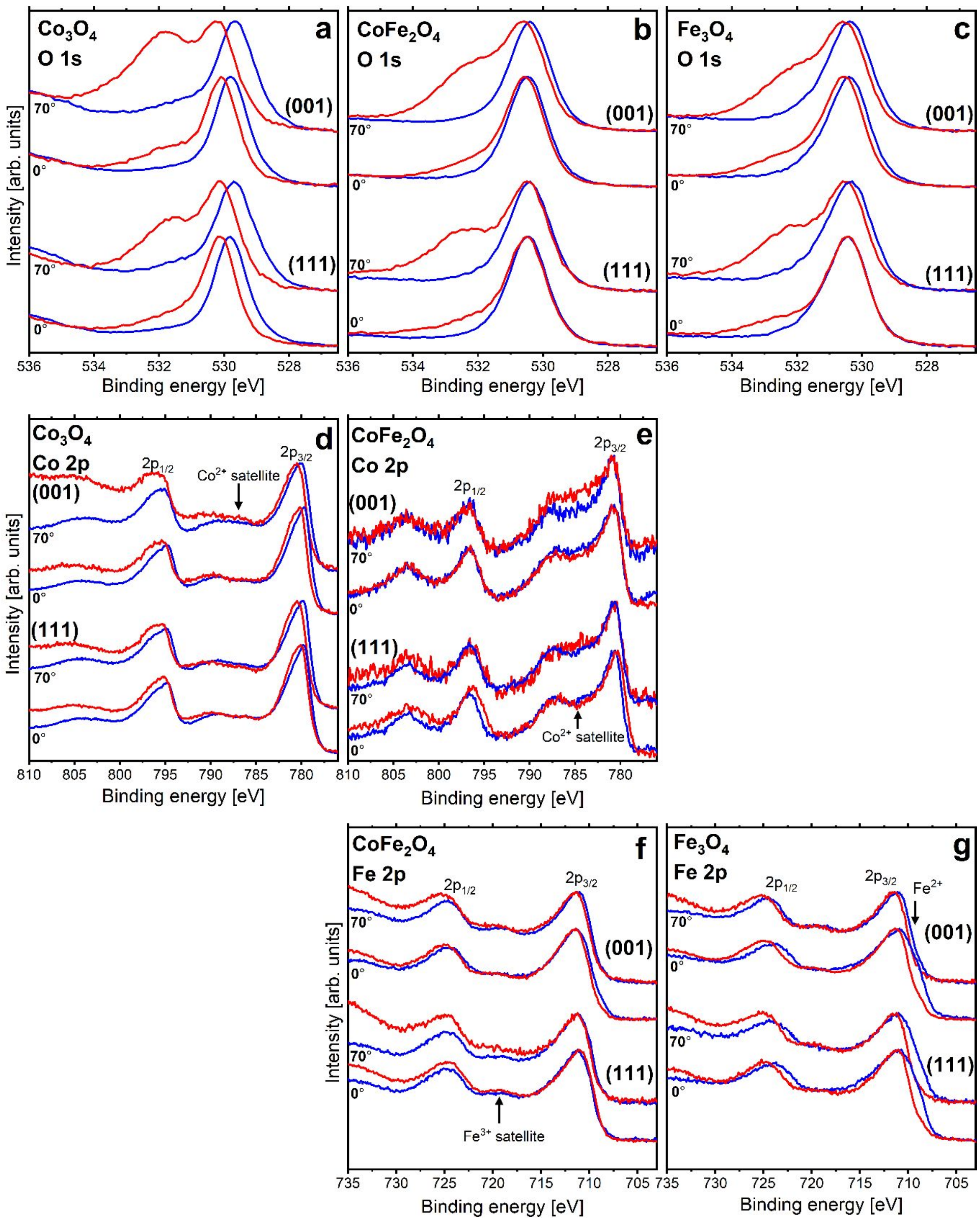

Figure S4 *– Normal (0°) and grazing (70°) incidence XPS spectra (Mg K_α radiation) from the Co_3O_4, $CoFe_2O_4$, and Fe_3O_4 thin films before (blue lines) and after (red lines) the OER experiments. The spectra were normalised so that the main peaks were of equal height.*

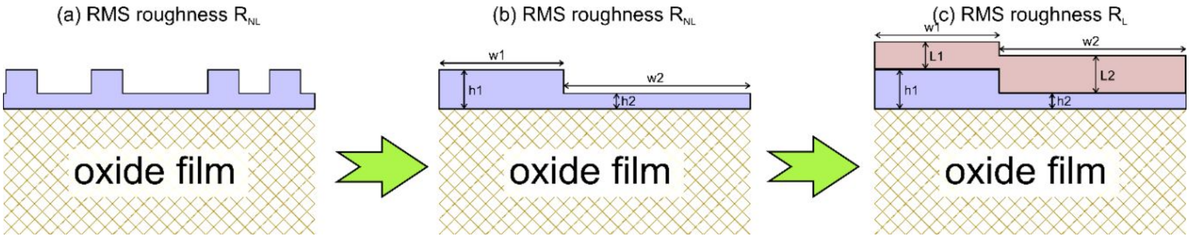

Figure S5 *– Estimation of the film roughness. For the model calculations a system with two height levels was assumed. The hatched area is the bulk of the oxide film and the blue area is the surface. The elevated structures in panel (a) (blue) were moved together to yield a surface with a single step, panel (b). The RMS roughness values are identical. Panel (c) illustrates the situation after the OER experiment where a disordered layer (red) covers the surface. The RMS roughness is now different.*

Layer thickness modelling computations

For the layer thickness modelling calculations, a system with two height levels as shown in panel (b) of Figure S5 was assumed. This was possible since the RMS roughness for the left and the right panels are identical. The thickness of the two overlayer areas (h1, h2) and the areas (w1, w2) were varied to find configurations with the RMS surface roughness listed in Table 1 for the specific system (before OER). A disordered layer was added, see panel (c) and the heights L1 and L2 were varied to find configurations with the RMS surface roughness (after OER) listed in Table 1 and the same XPS intensity ratio as listed in Table 2 or the same LEED spot intensity damping as listed in Table 2. Many different configurations with different average layer thickness were found. Table 2 lists the smallest values. To avoid the incorporation of structures not in agreement with the STM images, we have limited the height differences |h1-h2|, and |h1+L1-h2-L2| to values below 5nm. The scan range for the widths w1 and w2 was limited to 20 to 80% of the full area to avoid the formation of tall structures with a small area. There are many simplifications in the model and therefore, the results might not perfectly match the real situation. They are merely an indication of the role of the surface roughness when it is incorporated into the thickness computation.

The RMS surface roughness for the surface before OER was computed according to the standard formula for the RMS roughness (analogously for the surface after OER):

$$R_{RMS} = \sqrt{\frac{w1 \times (h1 - M)^2 + w2 \times (h2 - M)^2}{w1 + w2}} = \left|\frac{h1 - h2}{w1 + w2}\right| \sqrt{w1 \times w2}$$

$$M = \frac{h1 \times w1 + h2 \times w2}{w1 + w2}$$

M is the mean layer thickness.

For the calculations based on XPS intensity ratios, the intensity ratio of the respective O-1s peaks, $\frac{\mathrm{I(OH)}}{\mathrm{I(Substrate)}}$ in Figure 3, was used to determine the layer thickness. The intensities may be computed as

$$\mathrm{I(OH)} = \mathrm{P_{Layer}} \times \left(1 - e^{-\frac{D}{\lambda_{Layer} \times \cos\theta}}\right) \times \lambda_{Layer} \times \cos\theta$$

and

$$\mathrm{I(Substrate)} = \mathrm{P_{Substrate}} \times e^{-\frac{D}{\lambda_{SubstrateLayer} \times \cos\theta}} \times \lambda_{Substrate} \times \cos\theta$$

P_{Layer} and $P_{Substrate}$ are factors representing the photon flux, the elemental concentration, the photoionization cross section and the analyser transmission for the layer and for the substrate, respectively. D is the layer thickness, θ the electron exit angle relative to the surface normal and $\lambda_{Substrate}$, $\lambda_{SubstrateLayer}$, and λ_{Layer} are the inelastic mean free path lengths of electrons emitted in the substrate and travelling through the substrate, of electrons emitted in the substrate travelling through the layer, and of electrons emitted in the layer and travelling through the layer, respectively. For the modelling, the intensities for the two layers were summed up and the sum of the OH intensities was divided by the sum of the substrate intensities. The λ's were computed with the free Quases TPP2M IMFP program[1] for Co_3O_4. Much of the above equations is based on the equation for the attenuation of an electron beam travelling through matter, $A = e^{-\frac{H}{\lambda}}$, where H is the electron travel distance.

For the calculations based on LEED spot intensity damping, the damping of the LEED intensity was determined for several spots (the most intense ones) in the LEED patterns (Figure 2). For the intensity determination we used a self-written program which integrated the intensity in a circular area around the spots and subtracted a background determined from the intensity at the border of the integration circle. The damping factor A was calculated following the equation given above ($A = e^{-\frac{H}{\lambda}}$). For the travel distance H it was considered that the electrons have to travel twice (in and out) through the layer and that the outgoing electrons leave the sample at an angle. It is clear that the overlayer will affect the LEED pattern and that the λ's do not consider elastic diffuse scattering, which will also weaken the LEED spots. Furthermore, contaminations such as carbon or potassium would also weaken the LEED spots. Also, it may affect the outcome if the layer is not fully amorphous (especially if the crystalline part has the same periodicity as the substrate). Thus, this approach is also considered to have a significant inaccuracy. However, the XPS-based approach may not be sensitive to oxyhydroxide and bulk hydroxide, since their XPS intensities may be partially hidden by the oxide O1s bulk peak, while the LEED-based approach catches all material transformed during OER, provided that it leads to a disordered layer after OER.

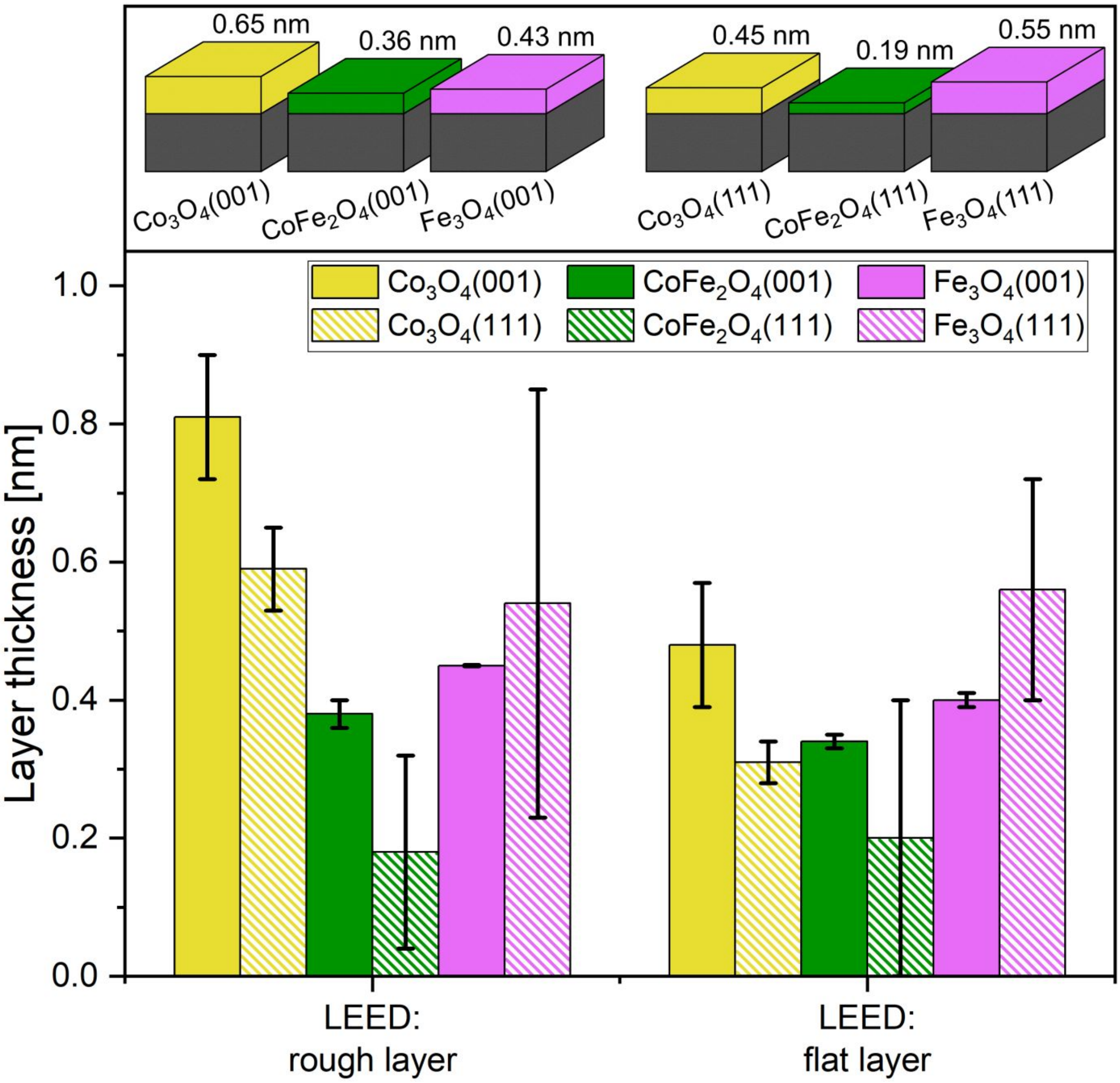

Figure S6 *- Bottom panel: skin layer thicknesses computed from LEED spot damping factors listed in the supporting Table S2. The smallest thicknesses compatible with the RMS roughness given in Table 1 and the layer thicknesses computed without consideration of the RMS roughness, just assuming a flat homogeneous layer on a flat substrate are shown. The top panel illustrates the different layer thicknesses; the given numbers are average thicknesses computed from the values in supporting Table S2, which lists the data in numerical form. The error bars are standard deviations derived from two datasets.*

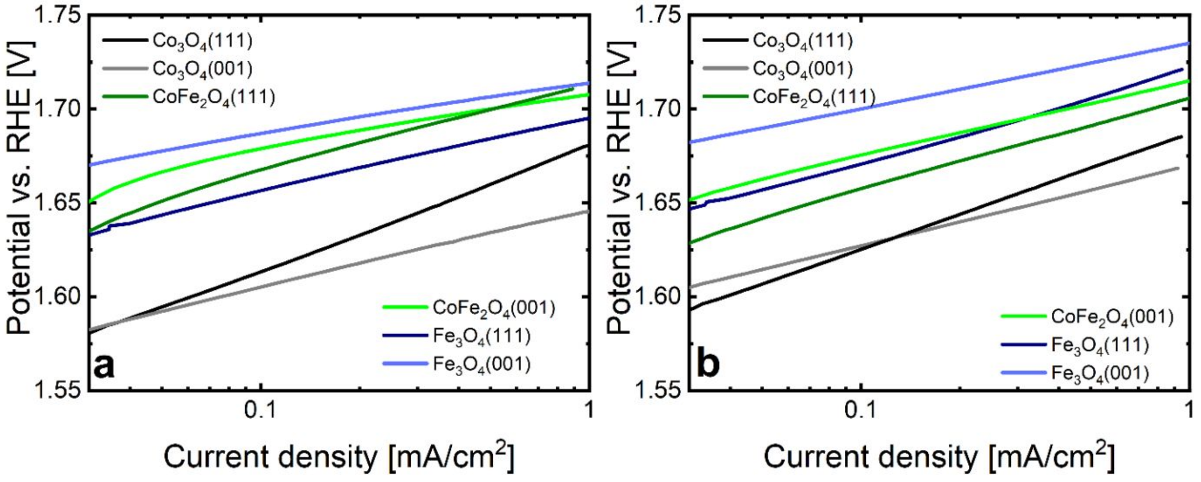

Figure S7 *– 'Tafel' plots for the LSV figures 6a (panel a Figure S7) and 6d (panel b in Figure S7) which show anodic LSV scans taken before and after OER at 1mA/cm^2 for two hours. The cobalt ferrite data are for films with an Fe:Co concentration ratio of 64:36 (determined with Al-k$_\alpha$ XPS for normal electron detection using the 2p peak intensities).*

Table S1 *– Electrochemical (Ω) resistances used for the IR correction of the LSV curves in Fig. 6.*

	**Before OER (2 hours)**		**After OER (2 hours)**	
	**(001)**	**(111)**	**(001)**	**(111)**
**Co_3O_4**	120.9	117.8	121.4	118.9
**$CoFe_2O_4$**	110	110	110	110
**Fe_3O_4**	116.9	92	122	90

Table S2 *– Flat film thickness and smallest mean skin layer thicknesses compatible with the determined RMS roughness values. The numbers are derived from XPS O1s intensity ratios (hydroxyl/bulk) or LEED spot damping factors. Also listed are the layer thicknesses computed without consideration of the RMS roughness, just assuming a flat homogeneous layer on a flat substrate.*

	**XPS**			**LEED**		
	**Intensity ratio**	**Rough layer thickness (nm)**	**Flat layer thickness (nm)**	**Damping factor**	**Rough layer thickness (nm)**	**Flat layer thickness (nm)**
**Co_3O_4(001)**	1.22±0.14	0.92±0.05	0.66±0.05	0.15±0.05	0.81±0.09	0.48±0.09
**Co_3O_4(111)**	0.82±0.08	0.71±0.03	0.50±0.03	0.28±0.01	0.59±0.06	0.31±0.03
**'(001)'-'(111)'**		0.21	0.16		0.22	0.17
**$CoFe_2O_4$(001)**	0.61±0.04	0.43±0.02	0.41±0.02	0.25±0.02	0.38±0.02	0.34±0.01
**$CoFe_2O_4$(111)**	0.58±0.11	0.50±0.21	0.39±0.06	0.57±0.31	0.18±0.14	0.20±0.20
**'(001)'-'(111)'**		-0.07	0.02		0.2	0.14
**Fe_3O_4(001)**	0.69±0.05	0.47±0.03	0.44±0.02	0.20±0.01	0.45±0.00	0.40±0.01
**Fe_3O_4(111)**	0.64±0.06	0.42±0.03	0.42±0.03	0.16±0.17	0.54±0.31	0.56±0.35
**'(001)'-'(111)'**		0.05	0.02		0.11	0.16

Table S3 *– 'Tafel' slopes in mV/decade. The listed numbers are averages, and the error margins are standard deviations computed with data from two datasets of identically prepared samples.*

	**Before OER (2 hours)**		**After OER (2 hours)**	
	**(001)**	**(111)**	**(001)**	**(111)**
**Co_3O_4**	41 ± 9	67 ± 10	43 ± 14	62 ± 4
**$CoFe_2O_4$**	30 ± 1	46 ± 4	42 ± 2	48 ± 0
**Fe_3O_4**	28 ± 2	39 ± 2	35 ± 32	51 ± 13

***Table S4** – Activity (O_2 molecules per second per nm^2) of the thin films at various stages of the experiment. 'anodic' and 'cathodic' refer to the scan direction. The error bars are standard deviation derived from two or more measurements and the data are averages. For $CoFe_2O_4$ films, error bars are not available since the prepared oxides had always slightly different Co:Fe compositions and therefore the data were not averaged in view of the strong impact of the composition on the OER reactivity.*

Composition	Facet	1st LSV anodic	1st LSV cathodic	Post OER LSV cathodic	Post OER LSV anodic
Co_3O_4	(001)	4.98 ± 1.23	4.56 ± 0.81	2.05 ± 0.37	3.03 ± 1.22
	(111)	2.82 ± 0.02	1.58 ± 0.28	0.73 ± 0.20	1.84 ± 0.06
$CoFe_2O_4$	(001)	0.33	0.36	0.48	0.23
	(111)	0.36	0.48	1.05	0.67
Fe_3O_4	(001)	0.20 ± 0.15	0.06 ± 0.00	0.12 ± 0.04	0.07 ± 0.05
	(111)	0.41 ± 0.00	0.50 ± 0.09	0.30 ± 0.11	0.20 ± 0.01

Electrochemical cell

The electrochemical cell and its components, such as electrolyte containers, tubing, and metal-free syringes, underwent a thorough cleaning process to ensure cleanliness. They were initially immersed in a solution of $KMnO_4$ for 24 hours to eliminate any carbon-containing compounds through oxidation. Following this, everything was rinsed with ultrapure water and then treated with a diluted piranha solution for a few minutes. Components resistant to elevated temperatures (i.e., everything except the FKM seal) were subsequently boiled in ultrapure water. To ensure the thorough removal of metal contamination, non-metal parts were left in a diluted HNO_3 solution overnight. A final rinse in ultrapure water preceded the assembly of the cell in a glass chamber filled with Ar gas for the electrochemical experiments.

Our study involved examining the electrochemical performance of the thin films within an electrochemical cell connected to the UHV chamber. Sample transfer from the UHV chamber to the electrochemical cell was possible without exposure to air. The process involved transferring the sample to a load-lock, which was purged with pure Ar gas, and then to an attached glass chamber also filled with Ar gas. A PTFE electrochemical cell was pressed against the sample, sealing a 6 mm diameter circular area on the surface (for details see Davis et al.[2]). For Co_3O_4 films, the sealing component of the cell was substituted with a fluorocarbon (FKM) piece to avoid damaging the soft Au single crystals. The electrolyte used was a 0.1 M KOH solution prepared by dissolving KOH pellets with high purity in ultrapure water. For studies involving Co_3O_4, this was further purified following a method by Burke et al.[3] to minimize Fe contamination. Before introduction into the cell, the electrolyte was saturated with Ar by bubbling the gas through it for at least 30 minutes. Electrochemical measurements were conducted using a Biologic SP240 potentiostat with a ramp speed of 5 mV/s for the voltammograms. A leakless Ag/AgCl reference electrode and a Pt mesh as the counter electrode were used. To prevent exposure to air or foreign metals, the electrolyte was introduced into the cell via metal-free syringes and sealed tubes. After electrochemistry, the

sample was rinsed with ultrapure water bubbled with Ar before being reintroduced into the load-lock chamber. The load-lock was then evacuated to UHV using a turbomolecular pump before further analysis in the UHV chamber for 'quasi in situ' examination.

Ultra-high vacuum chamber

The ultra-high vacuum chamber utilized for sample preparation and surface characterization maintained a base pressure of 4×10^{-11} mbar. It was equipped with facilities for X-ray photoelectron spectroscopy (XPS), low-energy electron diffraction (LEED), and scanning tunnelling microscopy (STM) at room temperature. The XPS setup included an X-ray source with Al and Mg anodes, and a hemispherical analyser, all from Omicron GmbH, Germany. Measurements were typically carried out with normal emission geometry (0° with respect to the surface normal) using Mg Kα radiation (1253.6 eV). In some cases, the surface sensitivity was enhanced by measuring at non-normal detection angles. A pass energy of 20 eV in constant analyser energy mode was employed. Binding energy calibration utilized the Fermi edge and the 4f peaks of a gold single crystal. Spectral analysis involved subtracting a Shirley background and fitting peaks using pseudo-Voigt functions via CasaXPS software[4]. An Ar^+ ion gun was employed for sample cleaning. The sample could be heated either by heat radiation or electron bombardment using a tungsten filament situated behind the sample. Temperature measurement was possible via a K-type thermocouple spot-welded to the substrate's side.

Substrate and thickness dependence of thin film OER

There are studies where an effect of the support material is reported for very thin oxide films or islands (for instance, Yeo and Bell[5] or Fester et al.[6]). If the film is very thin, maybe just one monolayer, then the thin film oxide properties are strongly affected by the interaction with the substrate. Thus, the oxide properties are substrate-dependent and different from those of thick films and bulk materials. In those cases, it is the interaction with the substrate that is responsible for the substrate-dependent reactivity. In our case, the studied thin films are significantly thicker than just a monolayer (ca 10 nm which is about 21 times the distance between equivalent layers in $Co_3O_4(111)$). Within the accuracy of the employed structural methods, STM and LEED, the film structure is identical to the 'bulk' structure, and therefore, we believe that such an effect can be largely ignored. We also confirmed that the layers are closed (i.e., the electrolyte cannot interact directly with the metallic substrate), and therefore, substrate-dependent effects resulting from a direct electrolyte-substrate interaction can also be ruled out.

However, it is known that band bending may result from the presence of the substrate-oxide interface. This could affect the reaction. We do not know the influence of this effect quantitatively since the vertical extension of the volume with significant band bending is not known. However, we expect this effect to be somewhat limited due to a significant density of defects in the film. This density is rather in the range of percent than in the per mill range, far beyond that of common industrially made semiconductors. We also note that there are more

sources for band bending in the system such as the oxide/oxyhydroxide interface and the electrolyte/oxyhydroxide interface, which might affect the electronic structures more than the band bending resulting from the substrate/oxide junction which is 10nm away from the surface.

References

(1) Tougaard, S. QUASES-IMFP-TPP2M Ver.3.0 (http://www.quases.com).
(2) Davis, E. M.; Bergmann, A.; Zhan, C.; Kuhlenbeck, H.; Cuenya, B. R. Comparative study of Co3O4(111), CoFe2O4(111), and Fe3O4(111) thin film electrocatalysts for the oxygen evolution reaction. *Nature Communications* **2023**, *14* (1), 4791. DOI: 10.1038/s41467-023-40461-0.
(3) Burke, M. S.; Kast, M. G.; Trotochaud, L.; Smith, A. M.; Boettcher, S. W. Cobalt–Iron (Oxy)hydroxide Oxygen Evolution Electrocatalysts: The Role of Structure and Composition on Activity, Stability, and Mechanism. *Journal of the American Chemical Society* **2015**, *137* (10), 3638-3648. DOI: 10.1021/jacs.5b00281.
(4) Fairley, N.; Fernandez, V.; Richard-Plouet, M.; Guillot-Deudon, C.; Walton, J.; Smith, E.; Flahaut, D.; Greiner, M.; Biesinger, M.; Tougaard, S.; et al. Systematic and collaborative approach to problem solving using X-ray photoelectron spectroscopy. *Applied Surface Science Advances* **2021**, *5*, 100112. DOI: https://doi.org/10.1016/j.apsadv.2021.100112.
(5) Yeo, B. S.; Bell, A. T. Enhanced Activity of Gold-Supported Cobalt Oxide for the Electrochemical Evolution of Oxygen. *Journal of the American Chemical Society* **2011**, *133* (14), 5587-5593. DOI: 10.1021/ja200559j.
(6) Fester, J.; Makoveev, A.; Grumelli, D.; Gutzler, R.; Sun, Z.; Rodríguez-Fernández, J.; Kern, K.; Lauritsen, J. V. The Structure of the Cobalt Oxide/Au Catalyst Interface in Electrochemical Water Splitting. *Angewandte Chemie International Edition* **2018**, *57* (37), 11893-11897, https://doi.org/10.1002/anie.201804417. DOI: https://doi.org/10.1002/anie.201804417 (acccessed 2023/02/27).
